# A TMED3-governed disulfidptosis-related diagnostic signature reveals tumor microenvironment remodeling in intrahepatic cholangiocarcinoma

**DOI:** 10.3389/fimmu.2026.1858117

**Published:** 2026-07-29

**Authors:** Wanjia Qiao, Yixiang He, Jing Li, Xiaohan Liu, Lingfang Zhang, Xin Bai, Yeying Wang, Jianming Tang

**Affiliations:** 1 The First Clinical Medical College of Lanzhou University, Lanzhou, China; 2 The First Hospital of Lanzhou University, Lanzhou, China; 3 Gansu Provincial Hospital, Lanzhou, China

**Keywords:** diagnostic model, disulfidptosis, intrahepatic cholangiocarcinoma, single-cell RNA sequencing, tumor microenvironment

## Abstract

**Background:**

Intrahepatic cholangiocarcinoma (ICC) is an aggressive malignancy with poor prognosis and limited treatment options. Disulfidptosis, a novel cell death pathway driven by disulfide bond accumulation, has emerged as a potential mechanism in cancer biology; however, its role in ICC remains unclear.

**Methods:**

We integrated single−cell RNA sequencing (GSE138709) with bulk transcriptomic datasets (TCGA−CHOL, GSE107943, GSE32225) to systematically characterize the ICC cellular landscape. Analyses included CNV inference, stemness scoring, disulfidptosis activity assessment, and cell−cell communication profiling. A diagnostic model was constructed using LASSO−logistic regression with 10−fold cross−validation and validated in independent cohorts. TME characterization, survival analysis, and drug−target screening were also performed. Experimental validation included HPA immunohistochemistry, qRT−PCR, and functional assays following TMED3 knockdown.

**Results:**

Seven major cell types were identified, with malignant cholangiocytes exhibiting high aneuploidy (74%), elevated stemness, upregulated disulfidptosis activity, and extensive communication via SPP1−CD44 and IGFBP3−TMEM219 networks. A five−gene signature (TMED3, TMEM184B, MAPK13, MFSD10, GRB7) demonstrated robust diagnostic performance. Survival analysis showed borderline prognostic value for TMED3 (adjusted HR = 2.37, P = 0.073), while TMEM184B emerged as an independent prognostic factor (adjusted HR = 4.79, P = 0.028). PPI and co−expression analyses established links between signature genes and disulfidptosis regulators. Functional experiments confirmed that TMED3 knockdown suppressed ICC cell proliferation, migration, and enhanced sensitivity to glucose deprivation−induced disulfidptosis. Network−based drug screening identified eight high−priority candidates for therapeutic repurposing.

**Conclusion:**

This study provides a comprehensive single−cell atlas of ICC, identifies TMED3 as a key regulator of a disulfidptosis−related diagnostic signature, and demonstrates its functional role in promoting ICC malignancy. The five−gene signature shows diagnostic and prognostic promise, and the drug screening offers preliminary leads for therapeutic repurposing, providing a foundation for precision diagnosis and targeted therapy in ICC.

## 1 Introduction

Cholangiocarcinoma (CCA), a malignant neoplasm originating from the biliary epithelium, is classified according to anatomical location into intrahepatic cholangiocarcinoma (iCCA), perihilar cholangiocarcinoma (pCCA), and distal cholangiocarcinoma (dCCA) (1). In recent years, the global incidence and mortality rates of iCCA have been on a continuous upward trend. As the second most prevalent primary liver cancer, iCCA constitutes approximately 10%–15% of all primary hepatic malignancies (2–4). Epidemiological data from 32 countries indicate a rising global mortality rate for iCCA (5). The prognosis remains extremely poor, with a 5-year survival rate of less than 20% (2, 6). Due to nonspecific early symptoms and the absence of reliable screening biomarkers, the majority of patients are diagnosed at an advanced stage, precluding curative surgical resection. For these advanced cases, the standard first-line chemotherapy regimen, namely gemcitabine plus cisplatin, provides only limited clinical benefit (7). Although recent advances in combination immunotherapy, such as pembrolizumab with gemcitabine-cisplatin, have demonstrated modest improvements in outcomes (8), there is still a critical need to develop more effective therapeutic strategies to improve survival.

The high heterogeneity of iCCA represents a major contributor to its therapeutic challenges. This heterogeneity manifests not only across patients (inter-tumoral heterogeneity) but also within individual tumor lesions (intra-tumoral heterogeneity) (9, 10). Conventional bulk transcriptome sequencing yields only averaged gene expression profiles from heterogeneous cell populations, thereby limiting the ability to resolve the diverse cellular constituents and their dynamic interactions within the tumor microenvironment (TME) (11).

In recent years, advances in single-cell RNA sequencing (scRNA-seq) technology have enabled the systematic dissection of the TME in iCCA at single-cell resolution, encompassing malignant and non-malignant epithelial cells, immune cells (including T cells, B cells, and macrophages), and stromal cells such as cancer-associated fibroblasts and endothelial cells (10, 12). Notably, studies have identified six distinct fibroblast subsets in iCCA, revealing critical interactions between malignant cells and CD146-positive vascular cancer-associated fibroblasts mediated by IL-6 signaling (10). Moreover, recent research has delineated two molecular subtypes of iCCA, namely S100P^+^ SPP1^−^ and S100P^−^ SPP1^+^, which are characterized by distinct tumor microenvironment compositions and clinical outcomes (13). The inference of somatic copy number variations (CNVs) from scRNA-seq data facilitates the direct identification of malignant cells at the single-cell level, offering novel insights into tumor clonal evolution.

Within the TME of iCCA, malignant cholangiocytes engage in complex molecular crosstalk with the surrounding stromal and immune cells, and this collective interaction promotes tumor progression. Among these interactions, cellular metabolic reprogramming has emerged as a hallmark of cancer. Disulfidptosis is a recently identified form of regulated cell death. It is induced by the abnormal accumulation of intracellular disulfide bonds, which leads to the aberrant cross-linking of actin cytoskeletal proteins and subsequent cytoskeletal collapse (14). This process is particularly prevalent in cancer cells exhibiting high glycolytic activity and elevated expression of SLC7A11 (solute carrier family 7 member 11). These cells rely on SLC7A11 to import extracellular cystine, which is subsequently reduced to cysteine through the consumption of NADPH (nicotinamide adenine dinucleotide phosphate) (15). Under conditions of NADPH deficiency or impaired utilization, cystine accumulates intracellularly, driving excessive disulfide bond formation in actin-associated proteins, disrupting the actin network, and ultimately triggering disulfidptosis (16). However, the role of disulfidptosis in the pathogenesis and progression of iCCA, particularly its activation status within specific malignant cholangiocyte subpopulations and its interplay with components of the TME, remains poorly understood.

In summary, it is of great significance to leverage scRNA-seq technology to comprehensively analyze the cellular composition, intercellular communication, and functional states of iCCA, with a focus on emerging biological processes such as disulfidptosis. This approach can contribute to elucidating the pathogenesis of iCCA and identifying novel therapeutic targets and diagnostic biomarkers. This study aims to comprehensively and systematically characterize the cellular landscape of iCCA through the integration of single-cell and bulk transcriptomic data. It also intends to identify key malignant cell populations, investigate their metabolic vulnerabilities, especially the susceptibility to disulfidptosis. Ultimately, it aims to develop molecular signatures with diagnostic and prognostic value, thereby providing a robust theoretical foundation and candidate targets for the precise diagnosis and treatment of iCCA.

## 2 Materials and methods

### 2.1 Data acquisition and preprocessing

Bulk RNA-seq expression profiles and corresponding clinical data for cholangiocarcinoma (CHOL) patients were obtained from The Cancer Genome Atlas (TCGA) database (https://portal.gdc.cancer.gov/). Only ICC samples and matched adjacent normal tissue samples were retained for downstream analysis. Additional gene expression datasets, GSE107943 and GSE32225, were downloaded from the Gene Expression Omnibus (GEO) database (https://www.ncbi.nlm.nih.gov/geo/).

For descriptive purposes and to facilitate candidate gene discovery, the three datasets (TCGA, GSE107943, and GSE32225) were integrated into a unified dataset after batch effect correction using the ComBat algorithm implemented in the sva R package (version 3.42.0), with sample status (tumor/adjacent normal) included as a covariate to preserve biological variation. This integrated dataset (referred to as the combined dataset) comprised 215 tumor samples and 42 adjacent normal tissue samples. Importantly, this dataset was used solely for descriptive analyses (e.g., ESTIMATE scoring, differential expression for candidate gene discovery) and was not used for model training or validation.

To verify the effectiveness of batch correction, principal component analysis (PCA) was performed on the combined expression matrix before and after ComBat correction, with samples colored by dataset origin. As shown in **Supplementary Figures 2A, B**, samples from the three datasets exhibited clear separation prior to correction, indicating substantial batch effects, while they became well-intermixed after correction, confirming successful removal of batch effects.

For model development, GSE107943 was used as the training set, while TCGA and GSE32225 served as two independent validation sets. No batch correction was applied across the training and validation sets to avoid data leakage. Each dataset was normalized separately prior to analysis: microarray datasets (GSE107943 and GSE32225) were log_2_-transformed and quantile-normalized, while TCGA RNA-seq raw counts were converted to log_2_(TPM + 1) to achieve a scale comparable to the microarray data.

### 2.2 Single-cell RNA sequencing data processing

The single-cell RNA sequencing (scRNA-seq) dataset GSE138709, containing ICC samples, was retrieved from the GEO database. This dataset includes eight samples: five tumor specimens and three matched adjacent normal tissues. Raw data were processed using the Seurat R package (version 5.1.0) for quality control and preprocessing. Mitochondrial gene content was calculated using the Percentage FeatureSet function (17). Cells were filtered based on the following criteria: (1) detection of more than 250 expressed genes; (2) mitochondrial gene percentage below 10%; (3) unique molecular identifier (UMI) counts exceeding 250; and (4) log10(GenesPerUMI) > 0.8, where GenesPerUMI is defined as nFeature_RNA divided by nCount_RNA. To avoid potential artifacts from doublets—two or more cells captured within the same droplet—we performed doublet detection and removal using the DoubletFinder package (version 3.0) prior to downstream analysis. Briefly, artificial doublets were generated from the processed Seurat object, and the proportion of artificial nearest neighbors (pANN) was calculated for each real cell following PCA dimensionality reduction. The parameters were set as follows: pN = 0.25, the optimal pK was determined using the bimodality coefficient method, the expected doublet rate was estimated as 0.8% per 1,000 cells loaded according to 10x Genomics guidelines, and the homotypic doublet proportion was adjusted using the modelHomotypic function. Cells predicted as doublets were excluded from all subsequent analyses. Detailed doublet statistics for each sample, including raw cell numbers, expected doublet rates, detected doublets, and post-filtering cell counts, are provided in **Supplementary Table 1**, and diagnostic plots (pANN score distributions and UMAP visualization of doublet vs. non-doublet cells) are shown in **Supplementary Figure 1**.

### 2.3 Batch effect correction and dimensionality reduction

Batch effects across samples were corrected using the Harmony integration method via the IntegrateLayers function in Seurat. Dimensionality reduction was performed through principal component analysis (PCA), with 30 principal components selected based on the elbow plot. Cell clustering was conducted using the FindNeighbors and FindClusters functions at a resolution of 0.2, yielding 13 distinct cell clusters. Visualization was carried out using Uniform Manifold Approximation and Projection (UMAP) and t-distributed Stochastic Neighbor Embedding (t-SNE).

### 2.4 Cell type annotation

**Figure 1:**
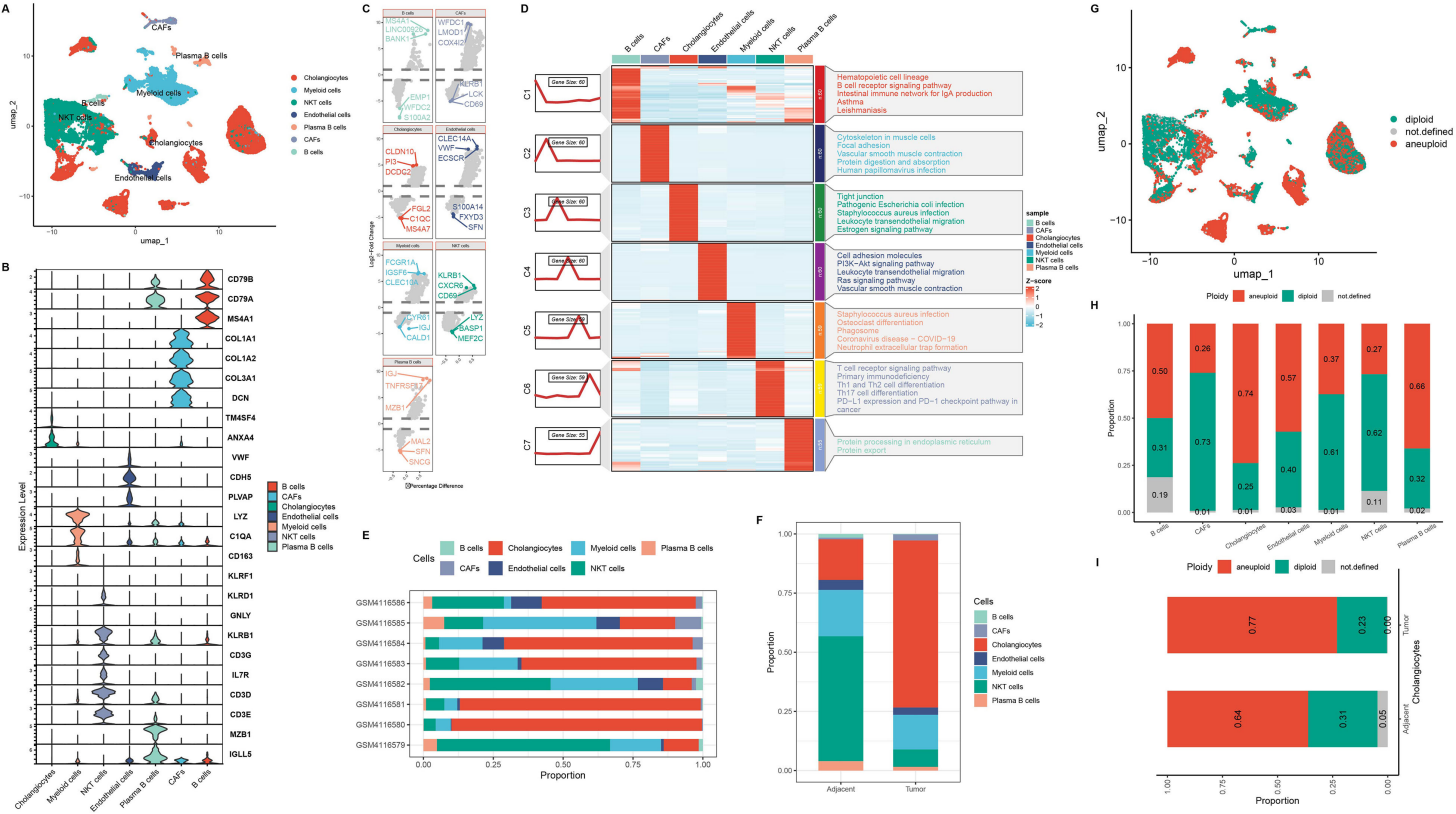
Single-cell transcriptomic analysis of the cellular composition and heterogeneity in the intrahepatic cholangiocarcinoma (iCCA) tumor microenvironment. **(A)** Schematic workflow illustrating quality control and integration of single-cell transcriptomic data from eight iCCA samples, including five tumor tissues and three paired adjacent non-tumor tissues. **(B)** UMAP visualization of single-cell clustering results, revealing 13 distinct transcriptional subpopulations. **(C)** Cell type annotation of the 13 subclusters based on canonical marker gene expression, classifying them into seven major cell types: cholangiocytes, T cells, B cells, macrophages, endothelial cells, fibroblasts, and other immune cells. Plasma B cells and myeloid sub−clusters are sub−types of the seven major cell lineages (see Methods). **(D)** Heatmap displaying the expression profiles of cell type–specific marker gene (rows: marker genes; columns: individual cells). **(E)** Bar graph depicting the proportional distribution of each cell type across the eight individual samples, highlighting inter-sample heterogeneity in cellular composition. **(F)** Comparative assessment of major cell type proportions between tumor and adjacent tissues, with notable alterations observed in cholangiocytes and NKT cells. **(G)** Schematic illustration of copy number variation (CNV) inference using inferCNV, with adjacent tissue cells serving as diploid reference controls. **(H)** Pie chart summarizing CNV status in annotated cholangiocytes, indicating that 74% exhibit aneuploid genomic profiles. **(I)** Quantitative comparison of aneuploidy frequency in cholangiocytes derived from tumor versus adjacent tissues.

Cell type annotation was conducted based on canonical marker genes derived from previous studies (10, 18–27). Seven major cell lineages were identified: cholangiocytes, T cells, NKT cells, B cells, myeloid cells, endothelial cells, and fibroblasts. NKT cells were defined as cells co−expressing CD3E (pan−T cell marker) and NCAM1 (CD56, a classical NK cell marker). Conventional T cells were identified as CD3E+NCAM1−, and NK cells as CD3E−NCAM1+.Finer sub−clusters (e.g., plasma B cells and myeloid sub−clusters shown in **Figure 1C**) are nested within these seven major lineages and are presented only to illustrate detailed heterogeneity; for quantitative comparisons and model construction, the major−type annotation was used. Differential expression analysis to identify cluster−specific marker genes was performed using the FindAllMarkers function in Seurat with thresholds of fold change (FC) > 2 and false discovery rate (FDR) < 0.05. Functional enrichment analysis of the marker genes was performed using the clusterProfiler R package to characterize biological processes and pathways associated with each cell type.

### 2.5 Copy number variation analysis

Copy number variation (CNV) inference was performed using the copyKAT algorithm to differentiate malignant from normal cells. Based on the analysis results, cells were classified as aneuploid (CNV-altered) or diploid (CNV-normal). The proportion of aneuploid cells was calculated for each cell type and compared between tumor and adjacent normal tissues.

### 2.6 Cell stemness analysis

Cellular stemness was assessed using the CytoTRACE algorithm, which infers cellular differentiation states from single-cell gene expression profiles. Stemness scores were computed for each cell, with higher values indicating greater stemness and lower differentiation potential. Comparisons of stemness levels were performed across cell types and between tumor and adjacent normal tissue groups.

### 2.7 Disulfidptosis scoring analysis

Disulfidptosis-related gene sets were curated from previously published literature (14, 28–30). To evaluate disulfidptosis activity at the single-cell level, we calculated pathway activity scores using six independent gene set enrichment algorithms implemented in the irGSEA R package: AUCell, UCell, singscore, ssGSEA, JASMINE, and viper (31, 32). Specifically, AUCell and UCell are ranking-based methods that assess pathway activity by evaluating gene expression ranks within each cell; singscore and ssGSEA are enrichment-based approaches that compute scores based on the relative expression levels of gene sets; and JASMINE and viper are network-based algorithms that infer pathway activity by integrating regulon information. These six algorithms were selected to provide a comprehensive and cross-validated assessment of disulfidptosis activity, each offering complementary strengths in scoring sensitivity and robustness. The scores obtained from each algorithm were then compared across different cell types to characterize cell-type-specific disulfidptosis activity patterns, as well as between tumor-derived and adjacent normal cholangiocytes to evaluate disulfidptosis activity changes associated with malignant transformation. Results from singscore, ssGSEA, and viper are presented in **Supplementary Figures 3**–**S5**, respectively.

### 2.8 Pathway scoring and transcription factor analysis

Pathway activity scores for immune, metabolic, signaling, and proliferation-related pathways were calculated using six gene set enrichment algorithms implemented in the irGSEA R package: AUCell, UCell, singscore, ssGSEA, JASMINE, and viper. Specifically, AUCell and UCell are ranking-based methods that assess pathway activity by evaluating gene expression ranks within each cell; singscore and ssGSEA are enrichment-based approaches that compute scores based on the relative expression levels of gene sets; and JASMINE and viper are network-based algorithms that infer pathway activity by integrating regulon information. These six algorithms were selected to provide a comprehensive and cross-validated assessment of pathway activity, each offering complementary strengths in scoring sensitivity and robustness. For brevity, the main figures present results from three representative algorithms (AUCell, UCell, and viper), while results from the remaining three algorithms (singscore, ssGSEA, and JASMINE) are provided in **Supplementary Figures 3**–**S5**. The SCENIC (Single-Cell Regulatory Network Inference and Clustering) algorithm was applied to infer transcription factor regulatory networks and compute transcription factor activity scores for each cell type.

### 2.9 Cell-cell communication analysis

Cell-cell communication analysis was performed using the CellChat R package (version 1.6.0) (33). The built-in CellChatDB.human database was used as the reference ligand-receptor interaction resource. Cell types with fewer than 10 cells were excluded to ensure statistical reliability. Significant ligand-receptor interactions were identified, and communication networks were visualized for key signaling pathways, including IGFBP, VTN, and SPP1.

### 2.10 Differential expression and functional enrichment analysis

Differential expression analysis between tumor and adjacent normal samples was performed using the limma R package on the combined dataset (TCGA, GSE107943, and GSE32225). Differentially expressed genes (DEGs) were identified using thresholds of | FC| > 1 and FDR < 0.05. KEGG pathway enrichment analysis and Gene Set Enrichment Analysis (GSEA) were conducted with the clusterProfiler R package. The ssGSEA method was applied to calculate cholangiocyte signature scores in bulk RNA-seq datasets.

### 2.11 Diagnostic model construction based on LASSO-Logistic regression

Using GSE107943 as the training set, we extracted the expression matrix of the five candidate genes (TMED3, TMEM184B, MAPK13, MFSD10, and GRB7) and constructed a diagnostic classification model. The model was established using a combination of LASSO (Least Absolute Shrinkage and Selection Operator) feature screening and Logistic regression. Disease samples were assigned a label of 1, and control samples were assigned a label of 0. To evaluate model stability, 10−fold cross−validation was performed within the training set. Model performance was assessed using receiver operating characteristic (ROC) curves and the area under the curve (AUC). The cross−validated AUC from the training set was used to evaluate internal stability, while TCGA−CHOL intrahepatic cholangiocarcinoma samples and GSE32225 served as independent validation cohorts. Predicted values were calculated based on the parameters established from the training set; the validation sets did not participate in any step of model training.

### 2.12 Tumor microenvironment analysis

Tumor microenvironment characteristics were assessed using the ESTIMATE algorithm to calculate StromalScore, ImmuneScore, and ESTIMATEScore for samples in the combined dataset (TCGA, GSE107943, and GSE32225) (34). This analysis was performed for descriptive purposes only and did not involve model training or validation. Spearman correlation analysis was performed to evaluate associations between the expression levels of the five diagnostic genes and the TME scores. Additionally, Correlation analysis was performed between TME scores and diagnostic model genes. Additionally, correlations between diagnostic genes and pathway activity scores related to immune, metabolic, signaling, and proliferation processes were evaluated. Associations with chemokines, receptors, and major histocompatibility complex (MHC) molecules were also examined. To account for multiple hypothesis testing, the Benjamini-Hochberg FDR correction was applied; only correlations with FDR < 0.05 were considered statistically significant.

### 2.13 Survival analysis

Survival analysis was performed using the TCGA-CHOL ICC cohort to evaluate the prognostic value of the five diagnostic genes. Progression-free interval (PFI) and disease-free interval (DFI) were used as primary endpoints. Patients were stratified into high and low-expression groups based on the optimal cut-point determined using the survminer package. Kaplan-Meier curves were generated, and survival differences between groups were compared using the log-rank test.

To further evaluate the independent prognostic value of each gene, univariable and multivariable Cox proportional hazards regression analyses were performed. The multivariable models were adjusted for available clinical covariates, including age and sex. Hazard ratios (HRs) with 95% confidence intervals (CIs) were calculated for each gene. The proportional hazards assumption was verified using Schoenfeld residuals. All statistical analyses were performed using the survival and survminer R packages. A two-sided P value < 0.05 was considered statistically significant. Given the limited sample size of the TCGA-CHOL cohort. these analyses should be considered exploratory, and the results are presented with their corresponding limitations in the Discussion.

### 2.14 Protein-protein interaction network and correlation analysis with disulfidptosis-related genes

To explore the potential functional associations between the five diagnostic genes (TMED3, TMEM184B, MAPK13, MFSD10, GRB7) and disulfidptosis-related genes, we performed protein-protein interaction (PPI) network analysis and correlation analysis.

PPI network analysis. The PPI network was constructed using the STRING database (version 11.0, https://string-db.org/) with a high-confidence interaction score threshold > 0.4. The network visualization was directly exported from the STRING website. The disulfidptosis-related gene set was defined by integrating multiple previously published studies, including the seminal work that first identified this cell death pathway, as well as subsequent investigations that expanded the core gene signatures (14, 28–30). The PPI network was generated by integrating the five diagnostic genes with the disulfidptosis-related gene set, and hub genes were identified based on degree centrality within the network.

Correlation analysis. To further validate the relationship between the five diagnostic genes and disulfidptosis-related genes, we performed Spearman correlation analysis using the TCGA-CHOL cohort. Spearman correlation coefficients (ρ) were calculated for each pair of diagnostic and disulfidptosis-related genes. Genes with |ρ| > 0.3 and P < 0.05 were considered significantly correlated, and the results were visualized as a heatmap.

Pathway enrichment analysis. KEGG pathway enrichment analysis was performed for the genes within the PPI network using the clusterProfiler R package, with a FDR < 0.05 set as the significance threshold.

### 2.15 Drug proximity analysis

To explore potential therapeutic agents targeting the biological network of TMED3, we performed a drug-target proximity analysis based on the TMED3-associated gene set. Using the combined dataset (TCGA, GSE107943, and GSE32225) for descriptive purposes only, we calculated Spearman correlations between TMED3 expression and all other genes. Genes with |ρ| > 0.3 and P < 0.05 were retained as significantly correlated genes, collectively defined as the TMED3-associated gene set (S, n = 1,905), which served as the seed for proximity analysis.

A high-confidence human PPI network was downloaded from the STRING database (version 11.0) with a combined confidence score threshold > 400. Drug target information was obtained from DrugBank (version 5.1.12). Only FDA-approved drugs and compounds that have entered clinical trials (Phase I-III) were retained. Endogenous metabolites (e.g., NADH, copper, zinc, glycine, ornithine, and their salts) were manually excluded based on DrugBank categories and chemical classification.

The network proximity between the TMED3-associated gene set S and a drug target set T was calculated using the average shortest path distance with a hub-gene penalty weight:


$$d(S,T)=\frac{1}{\left|T\right|}\sum_{t\in T}\begin{array}{c} \min\\ \text{s∈S} \end{array}(d(s,t)+\omega_{t})$$

where d(s,t) denotes the shortest path distance between nodes s and t in the PPI network. To account for hub genes that may artificially shorten network distances, a weight ω_t_ was introduced for target genes that already belong to S:


$$\omega_{t}=\{\begin{array}{c} -\ln(D_{t}+1),\;\text{if}\;t\in S\\ 0\;,\;\;\;\;\;\;\;\;\;\;\;\;\;\text{otherwise} \end{array}$$

Where D*_t_* is the degree of node *t* in the PPI network. The rationale for using -ln(D*_t_* + 1) is to down-weight highly connected hub genes that would otherwise dominate shortest-path calculations.

To assess statistical significance, a null distribution was generated by randomly sampling 10,000 protein sets R of the same size as T from the PPI network, with degree-preserving randomization to control for node connectivity. The standardized proximity Z-score was computed as:


$$z(S,T)=\frac{d(S,T)-\mu_{d(S,R)}}{\sigma_{d(S,R)}}$$

Where *μ_d(S,R)_* and *σ_d_*_(_*_S_*,*_R_*_)_ are the mean and standard deviation of the null distribution. Multiple hypothesis testing was corrected using the Benjamini-Hochberg FDR method. Drugs with FDR < 0.001 and a Z-score significantly lower than the null expectation (i.e., shorter distance) were considered candidate therapeutic agents. For each prioritized candidate, we manually curated its known mechanism of action and existing evidence in intrahepatic cholangiocarcinoma or hepatobiliary cancers (**Supplementary Table 2**).

### 2.16 *In vitro* cell culture

Human intrahepatic cholangiocarcinoma cell lines HuCCT1 and RBE, as well as the normal human biliary epithelial cell line HiBEC, were purchased from the Cell Bank of the Chinese Academy of Sciences (Shanghai, China). All cell lines were cultured in RPMI-1640 medium (Servicebio, China) supplemented with 10% fetal bovine serum (FBS; Cell-box, China) and 1% penicillin-streptomycin (Servicebio, China). Cells were maintained in a humidified incubator at 37 °C with 5% CO_2_. Passaging was performed every 2–3 days using 0.25% trypsin-EDTA (Servicebio, China) to maintain cells in the logarithmic growth phase.

### 2.17 Quantitative real-time PCR validation

To validate the bioinformatics analysis results, quantitative real-time PCR (qRT-PCR) was performed to examine the expression levels of candidate genes (MAPK13, MMP14, PAFAH1B3, TMED3, and TMEM184B). Total RNA was extracted from ICC cell lines (HuCCT1 and RBE) and normal biliary epithelial cells (HiBEC) using TRIzol reagent according to the manufacturer’s instructions. Complementary DNA (cDNA) was synthesized using the PrimeScript RT Reagent Kit. qRT-PCR was conducted on a QuantStudio 5 Real-Time PCR System with SYBR Green Premix. GAPDH was used as the internal reference gene for normalization. Relative gene expression levels were calculated using the 2^−ΔΔCt^ method. The PCR primer synthesis was performed by Tsingke Biotech Co., Ltd (Xi’an, China). The primer sequences are listed in **Supplementary Table 3**.

### 2.18 Protein expression analysis using the human protein atlas database

Protein expression data for the candidate genes (TMED3, TMEM184B, MAPK13, MFSD10 and GRB7) were obtained from the Human Protein Atlas (HPA) database (https://www.proteinatlas.org/). Based on immunohistochemistry (IHC) staining results available in the HPA database, protein expression levels and subcellular localization were evaluated in normal liver tissues and intrahepatic cholangiocarcinoma tissues. Semi-quantitative assessment of protein expression was performed using the HPA pathology scoring system (not detected, low, medium, high). Representative tissue section images were selected to visualize and compare protein expression patterns.

### 2.19 Lentiviral transduction and stable cell line construction

To establish stable TMED3-knockdown cell lines, lentiviral particles encoding short hairpin RNAs (shRNAs) targeting TMED3 (shTMED3-1, shTMED3-2, shTMED3-3) or a non-targeting negative control shRNA (NC) were constructed and packaged by Shanghai GeneChem Co., Ltd. (Shanghai, China). HuCCT1 and RBE cells were seeded in 6-well plates and infected when cell confluence reached 30%–50%. HuCCT1 cells were transduced with lentiviral particles at a multiplicity of infection (MOI) of 30, while RBE cells were transduced at an MOI of 20, in the presence of GeneChem infection enhancer solution (GeneChem, China). After 24 h of infection, the medium was replaced with fresh complete medium. Following 48 h of additional culture, stable transductants were selected using complete medium containing 2 μg/ml puromycin (Beyotime, China) for 7 days, with fresh selective medium changed every 2–3 days. Knockdown efficiency of TMED3 was confirmed by Western blot analysis prior to subsequent experiments.

### 2.20 Western blot analysis

Cells were lysed using RIPA lysis buffer (Servicebio, China) supplemented with protease and phosphatase inhibitors (Servicebio, China). Protein concentrations were determined using a BCA protein assay kit (Servicebio, China). Equal amounts of protein (20–30 μg) were separated by SDS-PAGE and transferred onto PVDF membranes (Millipore, USA). The membranes were blocked with 5% non-fat milk in TBST for 1 h at room temperature and subsequently incubated with primary antibodies against TMED3 (dilution 1:1000, Proteintech, China) and GAPDH (dilution 1:5000, Proteintech, China) overnight at 4 °C. After washing with TBST, the membranes were incubated with HRP-conjugated secondary antibodies (dilution 1:5000, Proteintech, China) for 1 h at room temperature. Protein bands were visualized using an enhanced chemiluminescence (ECL) detection system (Servicebio, China), and band intensities were quantified using ImageJ software.

### 2.21 Cell proliferation assay (CCK-8)

Cell proliferation was evaluated using the Cell Counting Kit-8 (CCK-8, Servicebio, China). Briefly, HuCCT1 and RBE cells with stable TMED3 knockdown or NC control were seeded into 96-well plates at a density of 3 × 10^3^ cells per well in 100 μl of culture medium. At indicated time points (0, 24, 48, and 72 h), 10 μl of CCK-8 solution was added to each well and incubated for 2 h at 37 °C. Absorbance was measured at 450 nm using a microplate reader (Bio-Tek, USA). Each experiment was performed in triplicate and repeated at least three times independently.

### 2.22 Colony formation assay

For colony formation assays, cells were trypsinized and seeded into 6-well plates at a density of 500 cells per well. Cells were cultured for 10–14 days with medium changed every 3 days until visible colonies appeared. Colonies were fixed with 4% paraformaldehyde for 15 min and stained with 0.1% crystal violet (Beyotime, China) for 15 min at room temperature. After washing with distilled water, colonies containing ≥50 cells were counted under a light microscope. All assays were performed in triplicate and repeated three times independently.

### 2.23 EdU incorporation assay

DNA synthesis was evaluated using the BeyoClick™ EdU-555 Cell Proliferation Kit (Beyotime, China) according to the manufacturer’s instructions. Cells were seeded into 24-well plates at a density of 1 × 10^5^ cells per well and cultured overnight. EdU working solution (final concentration 10 μM) was added to each well and incubated for 2 h at 37 °C. Cells were then fixed with 4% paraformaldehyde for 15 min and permeabilized with 0.5% Triton X-100 for 15 min. EdU incorporation was detected by adding Click reaction cocktail containing Alexa Fluor 555 azide for 30 min at room temperature in the dark. Nuclei were counterstained with Hoechst 33342 (5 μg/ml) for 10 min. Images were captured using a fluorescence microscope (Nikon, Japan), and at least five random fields per well were selected for counting EdU-positive cells. The EdU-positive rate was calculated as the percentage of EdU-positive cells relative to total Hoechst-positive cells.

### 2.24 Wound healing assay

Cell migration was assessed using a wound healing assay. HuCCT1 and RBE cells with stable TMED3 knockdown or NC control were seeded into 6-well plates and cultured to 90–95% confluence. A straight scratch was created across the cell monolayer using a sterile 200 μl pipette tip. After scratching, cells were washed twice with PBS to remove detached cells and then cultured in serum-free medium. Wound closure was monitored, and images were captured at 0 h and 24 h using an inverted microscope (Nikon, Japan). Wound areas were measured using ImageJ software, and the wound healing rate was calculated as: (initial wound area − remaining wound area)/initial wound area × 100%. Each experiment was performed in triplicate.

### 2.25 Transwell migration assay

Cell migration was further evaluated using Transwell chambers (Labselect, China, 8 μm pore size). Cells were serum-starved for 12 h, trypsinized, and resuspended in serum-free medium at a density of 5 × 10^4^ cells per 200 μl. The cell suspension (200 μl) was added to the upper chamber, while the lower chamber was filled with 600 μl of complete medium containing 20% FBS as a chemoattractant. After incubation at 37 °C for 24 h, non-migrating cells on the upper surface of the membrane were gently removed with a cotton swab. Migrated cells on the lower surface were fixed with 4% paraformaldehyde for 15 min and stained with 0.1% crystal violet for 15 min. Stained cells were counted under a light microscope in five randomly selected fields per well. All experiments were conducted in triplicate and repeated three times independently.

### 2.26 Glucose deprivation-induced disulfidptosis assay

To investigate the role of TMED3 in disulfidptosis, HuCCT1 and RBE cells with stable TMED3 knockdown or NC control were seeded into confocal dishes and cultured overnight. The medium was then replaced with glucose-free RPMI-1640 medium (Servicebio, China) supplemented with 10% dialyzed fetal bovine serum (Gibco, USA) to exclude exogenous glucose interference, and cells were cultured for 24 h to induce disulfidptosis. After treatment, cells were fixed with 4% paraformaldehyde for 15 min, washed three times with PBS, and incubated with Actin-Tracker Red-594 (Beyotime, China) for 60 min at room temperature in the dark. After three additional PBS washes, cells were mounted with DAPI-containing anti-fade mounting medium (Solarbio, China). Images were acquired using a confocal microscope (Nikon, Japan) under 60× water immersion objective. At least five random fields per dish were selected to observe and document characteristic morphological features of disulfidptosis, including F-actin organization, cell shrinkage, and membrane blebbing.

### 2.27 Statistical analysis

All statistical analyses were performed using R software (version 4.3.0). For single-cell data analysis, Wilcoxon rank-sum tests were used for comparisons between two groups, and Kruskal-Wallis tests followed by Dunn’s post-hoc tests were applied for multi-group comparisons. Correlation analyses were conducted using Spearman’s rank correlation coefficients. For all correlation analyses, multiple hypothesis testing was corrected using the Benjamini-Hochberg FDR method, and only correlations with FDR < 0.05 were considered statistically significant.

For differential expression analysis of bulk transcriptomic data, the limma package was employed, and genes with |log_2_FC| > 1 and FDR < 0.05 were considered differentially expressed. For survival analysis, Kaplan-Meier curves were generated, and survival differences were compared using the log-rank test. Univariable and multivariable Cox proportional hazards regression models were used to evaluate the independent prognostic value of each gene, with hazard ratios (HRs) and 95% confidence intervals (CIs) reported. The proportional hazards assumption was verified using Schoenfeld residuals.

For cell proliferation, migration, and other functional assays, data are presented as mean ± standard deviation (SD) from at least three independent experiments. Comparisons between two groups were performed using two-tailed Student’s t-tests, and comparisons among multiple groups were performed using one-way analysis of variance (ANOVA) followed by Dunnett’s post-hoc test. For time-course experiments (e.g., CCK-8 assays), two-way ANOVA was applied. A two-sided P value < 0.05 was considered statistically significant, with significance levels indicated as *P < 0.05, **P < 0.01, and ***P < 0.001.

## 3 Results

### 3.1 Single-cell RNA sequencing analysis reveals cellular heterogeneity and identifies malignant cells in ICC

To comprehensively investigate the molecular characteristics of ICC, transcriptomic expression profiles and clinical information were obtained from multiple public databases. The Cancer Genome Atlas (TCGA) provided cholangiocarcinoma expression data, from which intrahepatic cholangiocarcinoma samples and matched adjacent normal tissues were selectively retained. Additionally, two independent datasets, GSE107943 and GSE32225, were downloaded from the Gene Expression Omnibus (GEO) database. For descriptive purposes and to facilitate candidate gene discovery, the TCGA, GSE107943, and GSE32225 datasets were integrated into a unified dataset after batch effect correction, termed the combined dataset (215 tumor samples and 42 adjacent normal tissue samples). This dataset was not used for model training or validation.

To elucidate the cellular composition and transcriptional landscape of ICC at single-cell resolution, we analyzed the scRNA-seq dataset GSE138709 from the GEO database, which included eight samples (five tumor tissues and three adjacent normal tissues). Quality control, normalization, and batch effect correction were performed, followed by dimensionality reduction and clustering, resulting in 13 distinct cell clusters. These clusters were annotated into seven major cell types: cholangiocytes, hepatocytes, fibroblasts, endothelial cells, macrophages, natural killer T (NKT) cells, and B cells (**Figures 1A–C**). To characterize the molecular features of each cell type, specific marker genes were identified using thresholds of FC > 2 and FDR < 0.05 (**Figure 1D**; **Supplementary Table 4**). Functional enrichment analysis revealed that cholangiocyte marker genes were significantly enriched in pathways associated with liver development and bile acid metabolism, whereas marker genes of immune cells (e.g., T cells and B cells) were predominantly involved in immune response and cell activation processes (**Figure 1E**; **Supplementary Table 5**). Notably, the proportion of cholangiocytes was substantially higher in tumor tissues, while that of NKT cells was markedly reduced (**Figure 1F**), indicating alterations in both epithelial and immune compartments. Cell type-specific marker genes were further analyzed through KEGG pathway enrichment, revealing distinct functional signatures (**Figures 1G, H**).

To distinguish malignant cells, we employed copyKAT to infer CNVs. 74% of cholangiocytes exhibited aneuploidy, with 77% of cholangiocytes in tumor tissues classified as aneuploid (**Figure 1I**). This finding provides strong evidence that the majority of cholangiocytes in tumor tissues have undergone malignant transformation.

### 3.2 Characterization of cellular stemness and disulfidptosis activity

**Figure 2:**
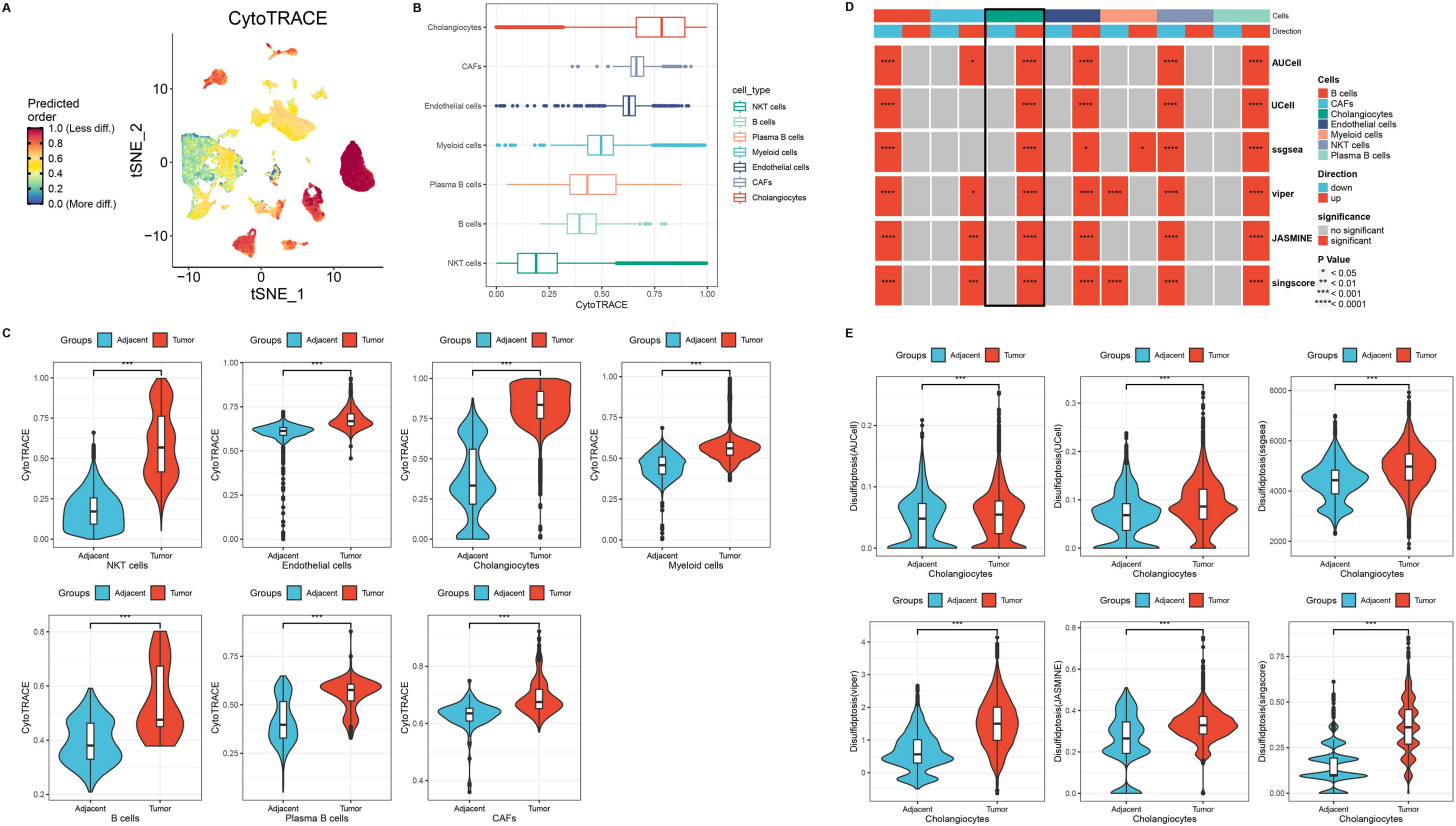
Functional characterization of malignant cholangiocytes and evaluation of disulfidptosis activity. **(A)** UMAP visualization of cell stemness scores inferred by the CytoTRACE algorithm, with color intensity reflecting the degree of stemness. **(B)** Box plot comparing stemness scores across major cell types, revealing significantly elevated stemness in cholangiocytes. **(C)** Comparative analysis of stemness scores between tumor and adjacent tissue–derived cells for each cell type, highlighting malignant cholangiocyte enrichment in stem-like properties. **(D)** Distribution of disulfidptosiscell death scores across cell types, as computed by six gene set enrichment algorithms (AUCell, Ucell, singscore, ssGSEA, JASMINE, and VIPER). **(E)** Comparison of disulfidptosiscell death activity in cholangiocytes derived from tumor versus adjacent tissues, based on integrated scoring from six computational methods.

To further characterize the developmental potential of different cell populations, we applied CytoTRACE to compute stemness scores at single-cell resolution. Comparative analysis revealed that cholangiocytes exhibited significantly higher stemness scores than other cell types, whereas immune cells—particularly NKT cells and B cells—displayed the lowest stemness levels (**Figures 2A, B**). These findings suggest that malignant cholangiocytes have acquired stem-like properties during tumorigenesis. Furthermore, we compared stemness scores between tumor and adjacent normal tissues across all cell types. Notably, all cell types in tumor tissues showed significantly higher stemness scores relative to their counterparts in adjacent normal tissues (**Figure 2C**). This observation implies that the malignant transformation of cholangiocytes may alter the developmental trajectory of surrounding cells, or that malignant cells have infiltrated neighboring cellular compartments.

Evaluation of disulfidptosis activity using six complementary scoring methods consistently revealed significant upregulation in cholangiocytes and downregulation in immune cells (**Figure 2D**). These findings suggest that disulfidptosis may play a crucial role in the development and progression of malignant cells while potentially attenuating immune responses. Notably, cholangiocytes from tumor tissues exhibited significantly higher disulfidptosis scores than those from normal tissues (**Figure 2E**), indicating its potential involvement in tumorigenesis.

### 3.3 Cell-specific pathway activity and transcription factor regulatory networks

**Figure 3:**
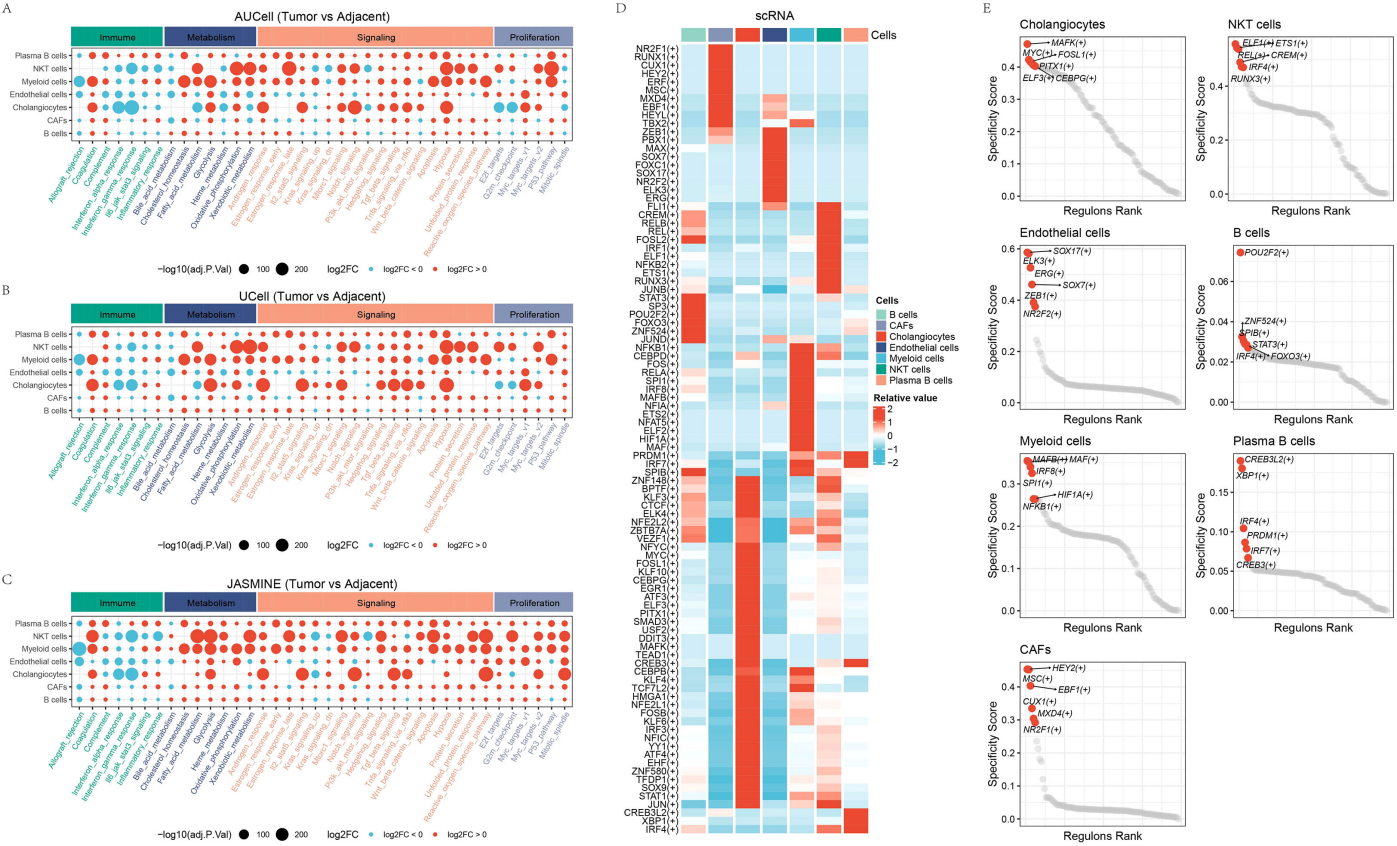
Single-cell profiling of pathway activities and transcription factor regulatory networks. **(A)** Dot plots showing differential analysis of various pathway activity scores across cell types between tumor and adjacent normal tissues, calculated using the AUCell algorithm.**(B)** Dot plots showing differential analysis of various pathway activity scores across cell types between tumor and adjacent normal tissues, calculated using the UCell algorithm. **(C)** Dot plots showing differential analysis of various pathway activity scores across cell types between tumor and adjacent normal tissues, calculated using the JASMINE algorithm. **(D)** Heatmap depicting cell type–specific transcription factor activity signatures. **(E)** Expression patterns of the top six most active transcription factors in each cell type, aligned with their inferred regulatory activities.

Given the potential heterogeneity in cellular processes across different cell types, we evaluated four categories of pathway activity scores—immune, metabolic, signaling, and proliferative pathways—using the irGSEA package. Comparative analysis between tumor and adjacent normal tissues revealed that cholangiocytes in tumor tissues exhibited elevated signaling and proliferation pathway scores, whereas immune cells, particularly NKT cells and B cells, showed reduced immune pathway scores in tumor tissues (**Figures 3A–C**).

Transcription factors (TFs) are crucial regulators that bind to specific DNA sequences to modulate gene transcription, thereby orchestrating various biological processes, including immune responses and developmental programs. We applied SCENIC to infer transcription factor activity at single-cell resolution. The analysis revealed distinct transcription factor activity profiles across different cell types (**Figure 3D**). Notably, the top six transcription factors with the highest specificity scores were identified in each cell type, uncovering substantial differences in transcriptional regulatory networks among cell populations (**Figure 3E**).

### 3.4 Cell-cell communication analysis reveals extensive crosstalk mediated by cholangiocytes

**Figure 4:**
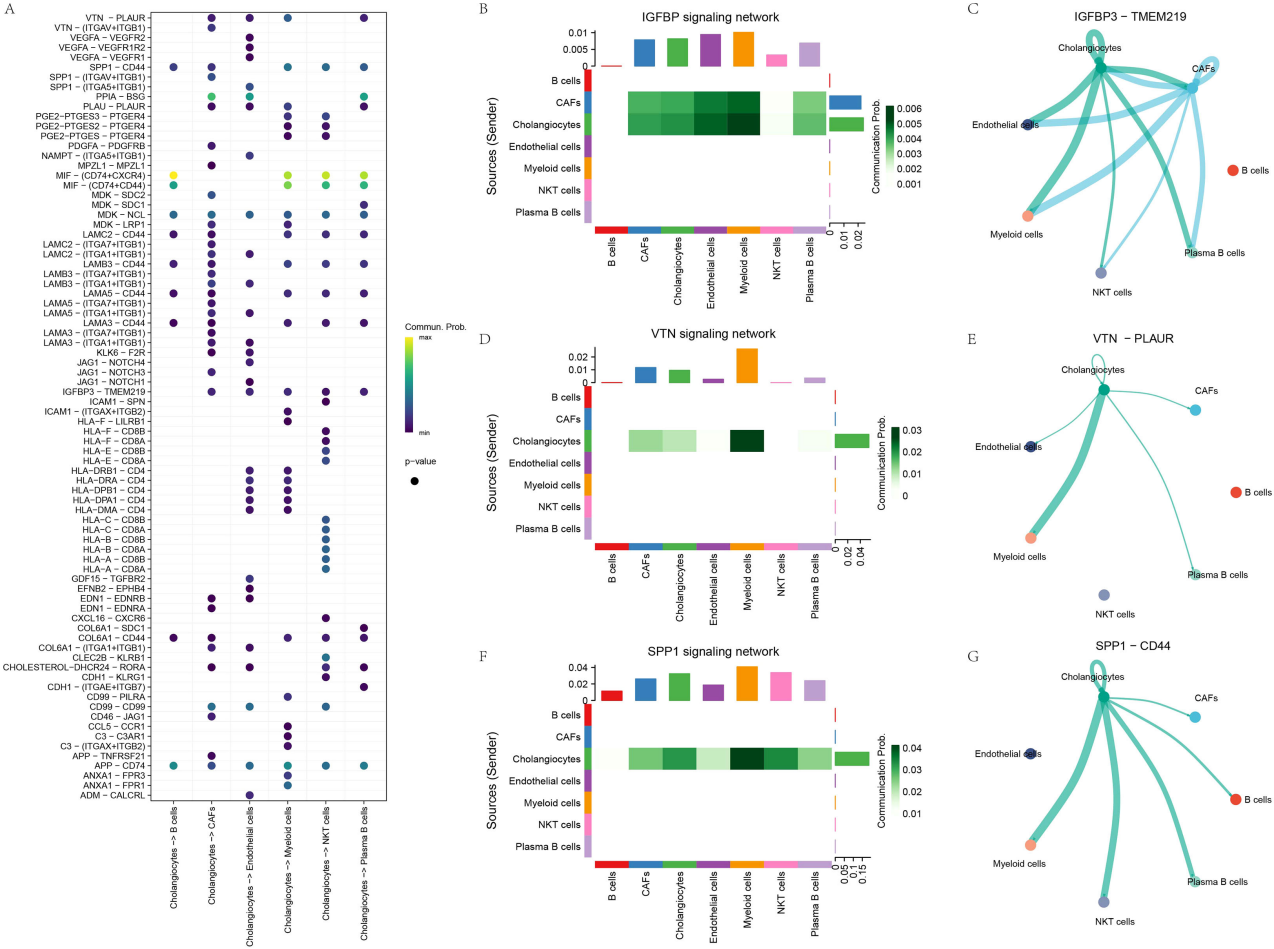
Intercellular communication network analysis in iCCA. **(A)** CellChat-based reconstruction of the intercellular communication network in iCCA, highlighting cholangiocytes as central signaling hubs. **(B)** Visualization of the IGFBP signaling network with identification of dominant ligand-receptor interactions. **(C)** Quantitative assessment of the IGFBP3–TMEM219 ligand-receptor interaction strength across cell types. **(D)** Visualization of the VTN signaling network, revealing key intercellular interaction pairs. **(E)** Interaction intensity analysis of the VTN -PLAUR ligand -receptor pair. **(F)** Visualization of the SPP1 signaling network, revealing key intercellular interaction pairs. **(G)** Interaction intensity analysis of the SPP1 -CD44 ligand -receptor pair.

Having identified seven major cell types with varying proportions across samples and observed significantly higher CNV frequencies in cholangiocytes compared to immune cells, we sought to investigate intercellular communication patterns, particularly those involving cholangiocytes. We applied CellChat (v1.6.0) with the built-in CellChatDB. human database as a reference to analyze cell–cell communication networks. The analysis revealed extensive interactions between cholangiocytes and other cell types (**Figure 4A**). Ligand–receptor interaction analysis further identified specific signaling pathways predominantly mediated by cholangiocytes. Cholangiocytes were found to communicate with cells in the tumor microenvironment through the IGFBP signaling network (ligand–receptor pair IGFBP3–TMEM219) (**Figures 4B, C**), the VTN signaling network (VTN–PLAUR) (**Figures 4D, E**), and the SPP1 signaling network (SPP1–CD44) (**Figures 4F, G**). These findings highlight the central role of cholangiocytes in orchestrating intercellular communication within the tumor microenvironment.

### 3.5 Validation in bulk RNA-seq datasets and differential gene expression analysis

**Figure 5:**
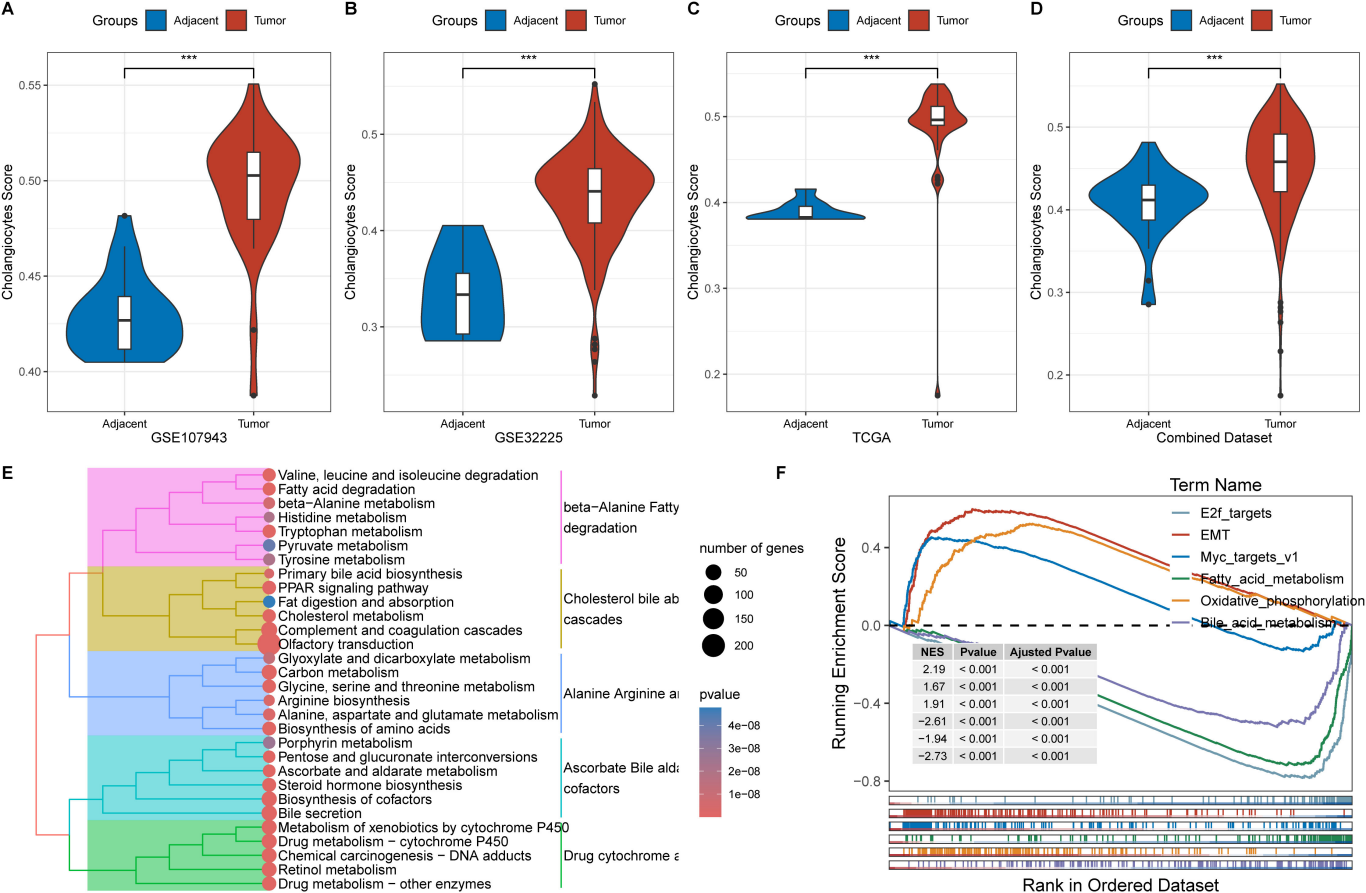
Validation of cholangiocyte signature and differential gene expression analysis. **(A–D)** Consistent elevation of cholangiocyte characteristic scores across four independent cohorts (TCGA, GSE107943, GSE32225, and Combined dataset), comparing tumor versus adjacent non-tumor tissues. **(E)** Bubble plot showing KEGG pathway enrichment results for differentially expressed genes in the Combined dataset, with significance levels and enrichment scores indicated. **(F)** GSEA enrichment map highlighting key signaling pathways significantly enriched in the Combined dataset.

Our single-cell analyses revealed that cholangiocytes have undergone malignant transformation, exhibit high stemness, display elevated disulfidptosis activity, and participate in extensive intercellular communication. To validate these findings using bulk RNA-seq data, we calculated cholangiocyte signature scores via ssGSEA based on upregulated marker genes identified from the single-cell analysis. Comparative analysis showed that cholangiocyte signature scores were significantly higher in tumor samples than in adjacent normal samples across all datasets, including TCGA, GSE107943, GSE32225, and the combined dataset (**Figures 5A–D**). These results confirm the consistency between single-cell and bulk transcriptomic data and support the conclusion that malignant transformation of cholangiocytes profoundly influences gene expression patterns.

Using the combined dataset, we performed differential gene expression analysis between tumor and adjacent normal samples. This analysis identified 1,498 DEGs, including 672 upregulated and 826 downregulated genes in tumor tissues. KEGG pathway enrichment analysis revealed that these DEGs were significantly enriched in the PPAR signaling pathway, retinol metabolism, fatty acid degradation, and drug metabolism–cytochrome P450 (**Figure 5E**). GSEA further showed that pathways related to E2F targets, epithelial–mesenchymal transition, and MYC targets were significantly enriched in tumor samples, whereas pathways associated with fatty acid metabolism, oxidative phosphorylation, and bile acid metabolism were enriched in adjacent normal samples (**Figure 5F**). These results highlight the metabolic reprogramming and activation of oncogenic pathways during ICC progression.

### 3.6 Machine learning−based construction of a diagnostic model

**Figure 6:**
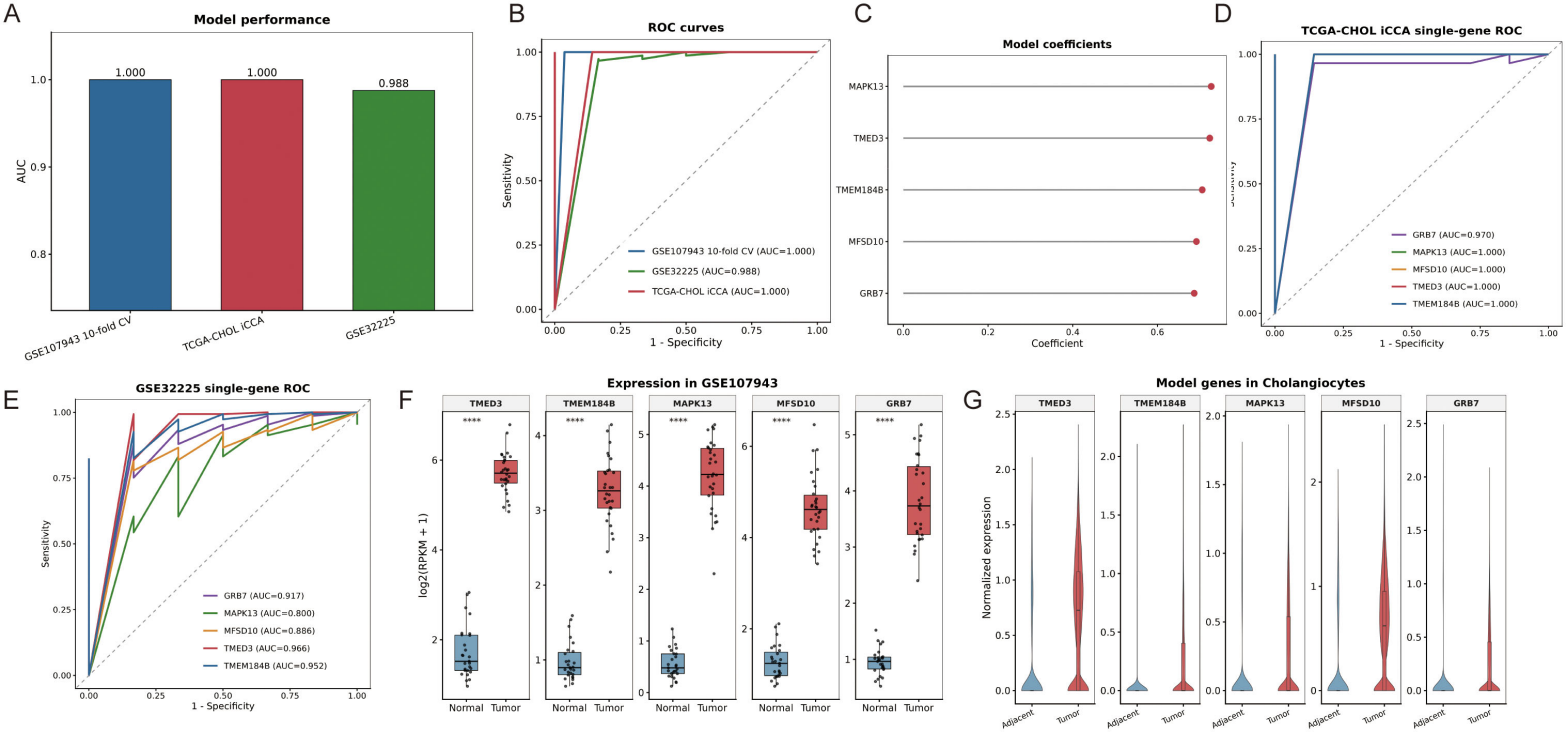
Construction and validation of the five−gene diagnostic signature based on LASSO−Logistic regression. **(A)** Diagnostic performance of the five−gene LASSO−Logistic regression model in the training set and independent validation cohorts. Bar plot showing the AUC values in the training set GSE107943 (10−fold cross−validation), the TCGA intrahepatic cholangiocarcinoma cohort, and the external validation set GSE32225, with corresponding AUCs of 1.000, 1.000, and 0.988, respectively. **(B)** ROC curves of the five−gene LASSO−Logistic regression model in the training set and independent validation cohorts. **(C)** LASSO regression coefficients of the five diagnostic genes (TMED3, TMEM184B, MAPK13, MFSD10, and GRB7) in the training set (GSE107943). **(D)** Individual ROC curves of the five diagnostic genes in the TCGA−CHOL iCCA validation cohort. **(E)** Individual ROC curves of the five diagnostic genes in the GSE32225 validation cohort. **(F)** Expression differences of the five diagnostic genes between tumor and adjacent normal tissues in the training set (GSE107943). **(G)** Expression differences of the five diagnostic genes between tumor−derived cholangiocytes and adjacent normal−derived cholangiocytes in the single−cell dataset (GSE138709). Data are presented as mean ± SD. ****P<0.0001.

To establish a robust diagnostic model for iCCA, we intersected differentially expressed genes with cholangiocyte marker genes to obtain a candidate gene set. The GSE107943 dataset was used as the training set, while TCGA−CHOL (iCCA samples only) and GSE32225 served as two independent validation cohorts. No samples from the training set were included in either validation set. Using GSE107943 as the training set, we extracted the expression matrix of five candidate genes (TMED3, TMEM184B, MAPK13, MFSD10, and GRB7) and constructed a diagnostic classification model combining LASSO feature selection with logistic regression. To evaluate internal robustness, 10−fold cross−validation was performed within the training set. The model achieved a high mean cross−validated AUC in the training set and demonstrated excellent diagnostic performance in both independent validation cohorts (**Figure 6A**). The ROC curves for the training set are shown in **Figure 6B**.

In the training set GSE107943, we presented the expression profiles of the selected genes and the LASSO regression coefficients of the final model (**Figure 6C**). As shown in **Figure 6C**, among the five diagnostic genes, MAPK13 exhibited the highest regression coefficient (0.726), followed by TMED3 (0.723), TMEM184B (0.705), and MFSD10 (0.692), while GRB7 showed the lowest coefficient (0.686), indicating that MAPK13 contributed the most to the diagnostic risk, whereas GRB7 contributed the least. ROC curve analysis was performed for each of the five diagnostic genes in the two independent validation cohorts. All five genes exhibited high individual AUC values in the TCGA−CHOL iCCA cohort (range: 0.970–1.000) (**Figure 6D**) and the GSE32225 cohort (range: 0.800–0.966) (**Figure 6E**).

In the GSE107943 training set, expression analysis confirmed that all five genes were significantly upregulated in tumor tissues compared with adjacent normal tissues (**Figure 6F**). To further validate these findings at the single−cell level, we analyzed the GSE138709 single−cell dataset, which revealed significantly higher expression of these five genes in tumor−derived cholangiocytes than in adjacent normal−derived cholangiocytes (**Figure 6G**), further supporting their association with iCCA malignant transformation.

### 3.7 Prognostic value assessment of the diagnostic genes

**Figure 7:**
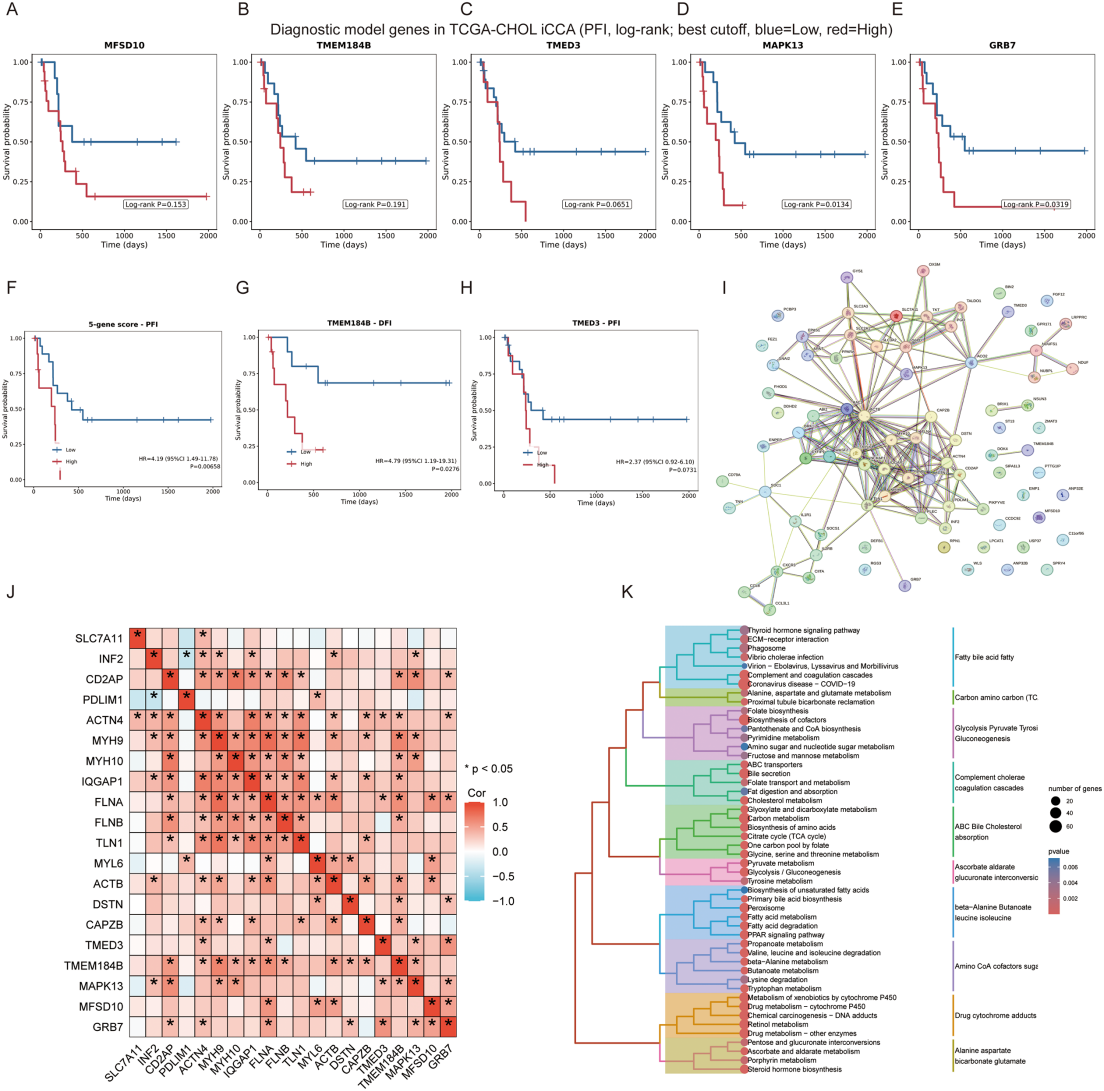
Prognostic evaluation, Cox regression analysis, drug-target proximity analysis, and exploration of disulfidptosis-related mechanisms of the diagnostic genes. **(A–E)** Kaplan-Meier curves showing progression-free interval differences between high- and low-expression groups of MFSD10, TMEM184B, TMED3, MAPK13, and GRB7 in the TCGA-CHOL intrahepatic cholangiocarcinoma cohort. **(F)** Kaplan-Meier curve of progression-free interval (PFI) stratified by the five-gene signature risk score. **(G)** Kaplan-Meier curve of disease-free interval (DFI) stratified by TMEM184B expression level. **(H)** Kaplan-Meier curve of progression-free interval (PFI) stratified by TMED3 expression level. **(I)** Protein-protein interaction network of the five diagnostic genes (TMED3, TMEM184B, MAPK13, MFSD10, GRB7) and disulfidptosis-related genes. **(J)** Heatmap showing expression correlations between the five diagnostic genes and core disulfidptosis gene sets in the TCGA-CHOL cohort. **(K)** Bubble plot of KEGG pathway enrichment analysis for TMED3-correlated genes. Data are presented as mean ± SD. *P < 0.05 vs. low-expression group (log-rank test for Kaplan-Meier curve comparisons; univariable and multivariable Cox regression for prognostic evaluation; Fisher’s exact test for pathway enrichment analysis).

To evaluate the prognostic value of the five diagnostic genes (TMED3, TMEM184B, MAPK13, MFSD10, and GRB7), we performed Kaplan-Meier survival analysis using progression-free interval (PFI) as the primary endpoint in the TCGA-CHOL iCCA cohort. Patients were stratified into high- and low-expression groups based on the optimal cut-point determined for each gene, and survival differences between groups were compared using the log-rank test. The results demonstrated that all five genes stratified patients into high- and low-risk groups, with high expression associated with worse prognosis (**Figures 7A–E**).

To further evaluate the independent prognostic value of each gene, univariable and multivariable Cox proportional hazards regression analyses were performed. Patients were stratified into high- and low-expression groups based on the optimal cut-point for each gene, with the low-expression group serving as the reference. Multivariable Cox regression analysis revealed that the combined five-gene risk score was significantly associated with PFI (HR = 4.19, 95% CI: 1.49–11.78, P = 0.0066) (**Figure 7F**). After adjusting for covariates, the TMEM184B high-expression group was significantly associated with disease-free interval (DFI) (HR = 4.79, 95% CI: 1.09–19.31, P = 0.028) (**Figure 7G**), identifying TMEM184B as an independent prognostic factor for iCCA. In contrast, the TMED3 high-expression group showed a borderline association with PFI (HR = 2.37, 95% CI: 0.92–6.10, P = 0.073) (**Figure 7H**), suggesting potential prognostic value that did not reach statistical significance, likely due to limited sample size. Detailed results of the univariable and multivariable Cox regression analyses are provided in **Supplementary Table 6**.

To assess the clinical utility of the model, decision curve analysis (DCA) and calibration curve analysis were performed in the TCGA-CHOL iCCA cohort (**Supplementary Figure 6**). The DCA demonstrated that the five-gene diagnostic signature provided positive net clinical benefit across a wide range of threshold probabilities, indicating favorable clinical applicability of the model. The calibration curve showed good agreement between predicted probabilities and observed outcomes, suggesting that the model was well-calibrated.

### 3.8 Drug-target proximity analysis and exploration of disulfidptosis-related mechanisms

To investigate the potential association between the core genes and disulfidptosis, we performed PPI network analysis by integrating the five diagnostic genes (TMED3, TMEM184B, MAPK13, MFSD10, and GRB7) with previously reported disulfidptosis-related gene sets (**Figure 7I**). The analysis revealed direct protein interactions between TMED3 and ACO2, MAPK13 and ACTB/SLC2A1, and GRB7 and TLN1, suggesting that these genes may participate in disulfidptosis-related biological processes through coordinated regulation.

To further validate these findings, we evaluated the expression correlations between the five diagnostic genes and the disulfidptosis-related gene set (including SLC7A11, INF2, MYH9, etc.) in the TCGA-CHOL cholangiocarcinoma cohort. Spearman correlation analysis demonstrated that TMED3, MAPK13, and TMEM184B were significantly positively correlated with most disulfidptosis core genes (|R| > 0.3, P < 0.05) (**Figure 7J**), indicating a potential transcriptional synergy between these diagnostic genes and disulfidptosis regulatory pathways.

To explore the therapeutic potential of TMED3, we performed drug-target proximity analysis based on the combined dataset (TCGA, GSE107943, and GSE32225). Genes significantly co-expressed with TMED3 were identified via Spearman correlation analysis (|R| > 0.3, P < 0.05) and served as the seed set for subsequent analysis. Following established methods, we calculated network proximity scores between drug targets and the seed gene set using DrugBank target annotations and the STRING protein-protein interaction network. A reference distribution was generated through 10,000 random permutations, and multiple hypothesis testing correction was applied, identifying 23 candidate drugs (FDR < 0.001). Endogenous metabolites (e.g., NADH, copper, zinc, zinc acetate, zinc chloride, zinc sulfate, glycine, ornithine, and their derivatives) were manually excluded, and the drug scope was restricted to FDA-approved drugs and compounds that have entered clinical trials. This filtering process reduced the candidate list from 23 to 8 high-priority drugs with therapeutic potential (**Table 1**).

**Table 1:** Candidate therapeutic agents with significant proximity to the TMED3-correlated gene set.

Drug_id	Drug_name	Distances	p value	FDR
DB12307	Foretinib	-6.1918	1.36E-08	7.44E-05
DB01213	Fomepizole	-5.04058	7.37E-12	4.04E-08
DB00951	Isoniazid	-4.96327	2.07E-08	0.000113
DB03516	Eniluracil	-4.76742	6.27E-08	0.000343
DB00642	Pemetrexed	-4.12782	8.75E-09	4.79E-05
DB00048	Collagenase clostridium histolyticum	-3.91572	3.81E-08	0.000209
DB08437	Puromycin	-2.64233	3.73E-10	2.05E-06
DB11638	Artenimol	-1.11695	2.73E-16	1.50E-12

Functional enrichment analysis of TMED3-correlated genes revealed significant enrichment in ECM-receptor interaction, PPAR signaling pathway, cholesterol metabolism, drug metabolism (other enzymes), carbon metabolism, and citrate cycle (TCA cycle) (**Figure 7K**). These findings provide potential therapeutic targets and molecular pathway insights into TMED3-mediated oncogenic mechanisms in ICC. Notably, the drug prediction results presented in this section are exploratory in nature, and the actual efficacy of the candidate drugs requires further validation through *in vitro* and *in vivo* experiments.

### 3.9 The diagnostic gene signature is associated with the tumor microenvironment and key pathways

**Figure 8:**
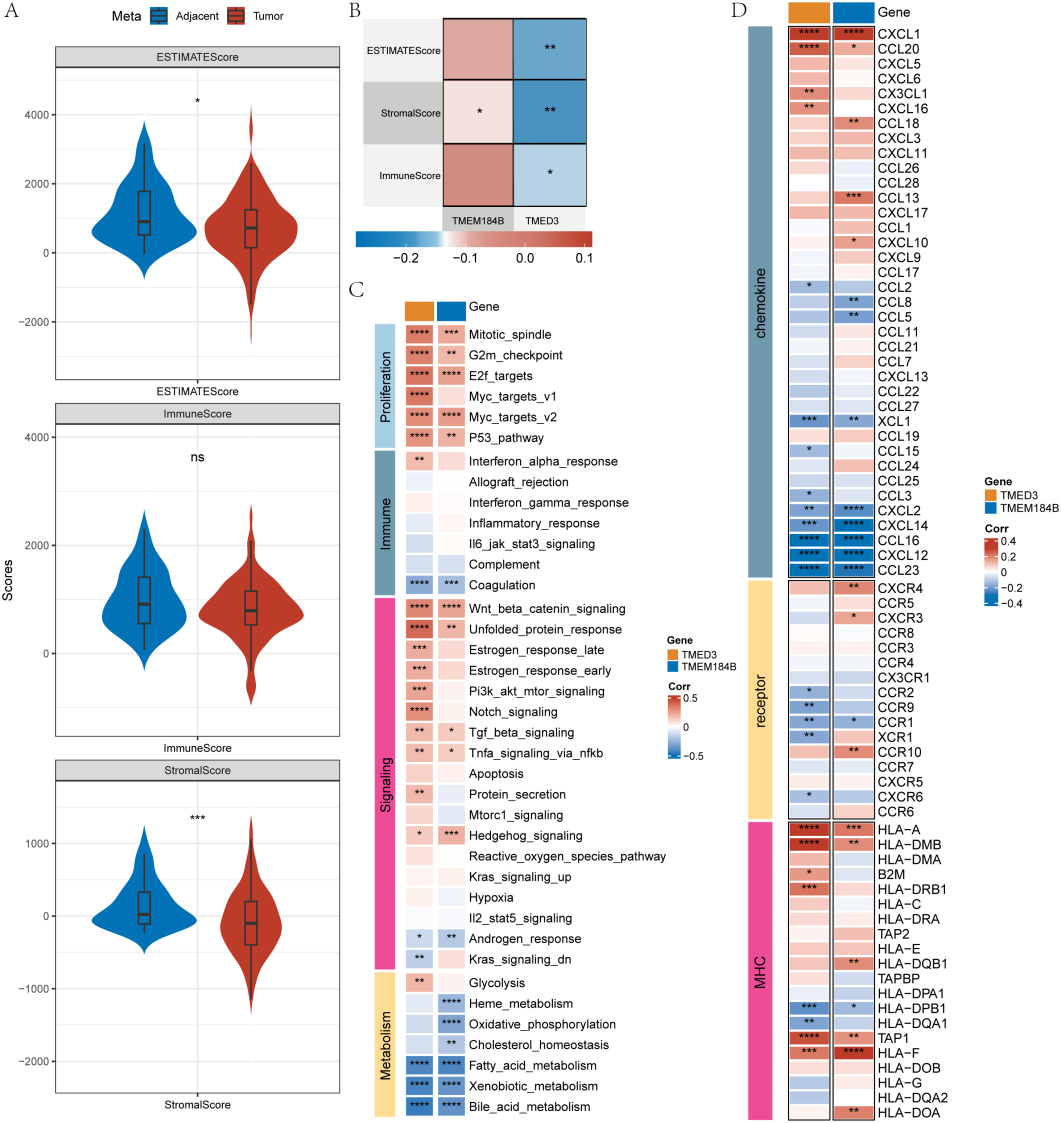
Association of the diagnostic gene signature with the tumor microenvironment and key pathways. **(A)** ESTIMATE algorithm evaluation of StromalScore, ImmuneScore, and ESTIMATEScore between tumor and adjacent normal samples in the combined dataset (TCGA, GSE107943, and GSE32225). **(B)** Correlation analysis between the diagnostic genes (TMED3 and TMEM184B) and ESTIMATE scores. **(C)** Correlation analysis between the diagnostic genes (TMED3 and TMEM184B) and four categories of pathway activity scores, including immune, metabolic, signaling, and proliferation pathways. **(D)** Correlation analysis between the diagnostic genes (TMED3 and TMEM184B) and three categories of immune−related genes, including chemokines, receptors, and MHC molecules. ESTIMATE analysis was performed for descriptive purposes only and did not involve model training or validation. Correlation analyses were performed using Spearman’s rank correlation coefficients, followed by Benjamini−Hochberg FDR correction, *P < 0.05, **P < 0.01, ***P < 0.001, ****P < 0.0001.

To characterize the tumor microenvironment, we applied the ESTIMATE algorithm to the combined dataset (TCGA, GSE107943, and GSE32225) to calculate StromalScore, ImmuneScore, and ESTIMATEScore. This analysis was performed for descriptive purposes only and did not involve model training or validation. Comparative analysis revealed that StromalScore and ESTIMATEScore were significantly higher in adjacent normal samples than in tumor samples, while ImmuneScore showed a similar trend without reaching statistical significance (**Figure 8A**). After Benjamini-Hochberg FDR correction (q < 0.05), TMED3 remained significantly negatively correlated with all three ESTIMATE scores, while TMEM184B showed a negative correlation trend that did not reach the FDR-adjusted significance threshold (**Figure 8B**).

We further assessed the correlations between the diagnostic genes (TMED3 and TMEM184B) and four categories of pathway activity scores, including immune, metabolic, signaling, and proliferation pathways. After FDR correction (q < 0.05), TMED3 and TMEM184B showed significant positive correlations with proliferation and signaling pathways, and significant negative correlations with metabolic pathways (**Figure 8C**), consistent with our single-cell sequencing findings. Additionally, TMED3 and TMEM184B exhibited significant correlations (FDR < 0.05) with three categories of immune-related genes, including chemokines, receptors, and MHC molecules (35) (**Figure 8D**).

Collectively, these findings suggest that the diagnostic gene signature may contribute to the malignant progression of cholangiocarcinoma by promoting tumor microenvironment remodeling, enhancing proliferative signaling, and suppressing metabolic pathways.

### 3.10 Experimental validation of candidate gene expression in cholangiocarcinoma

**Figure 9:**
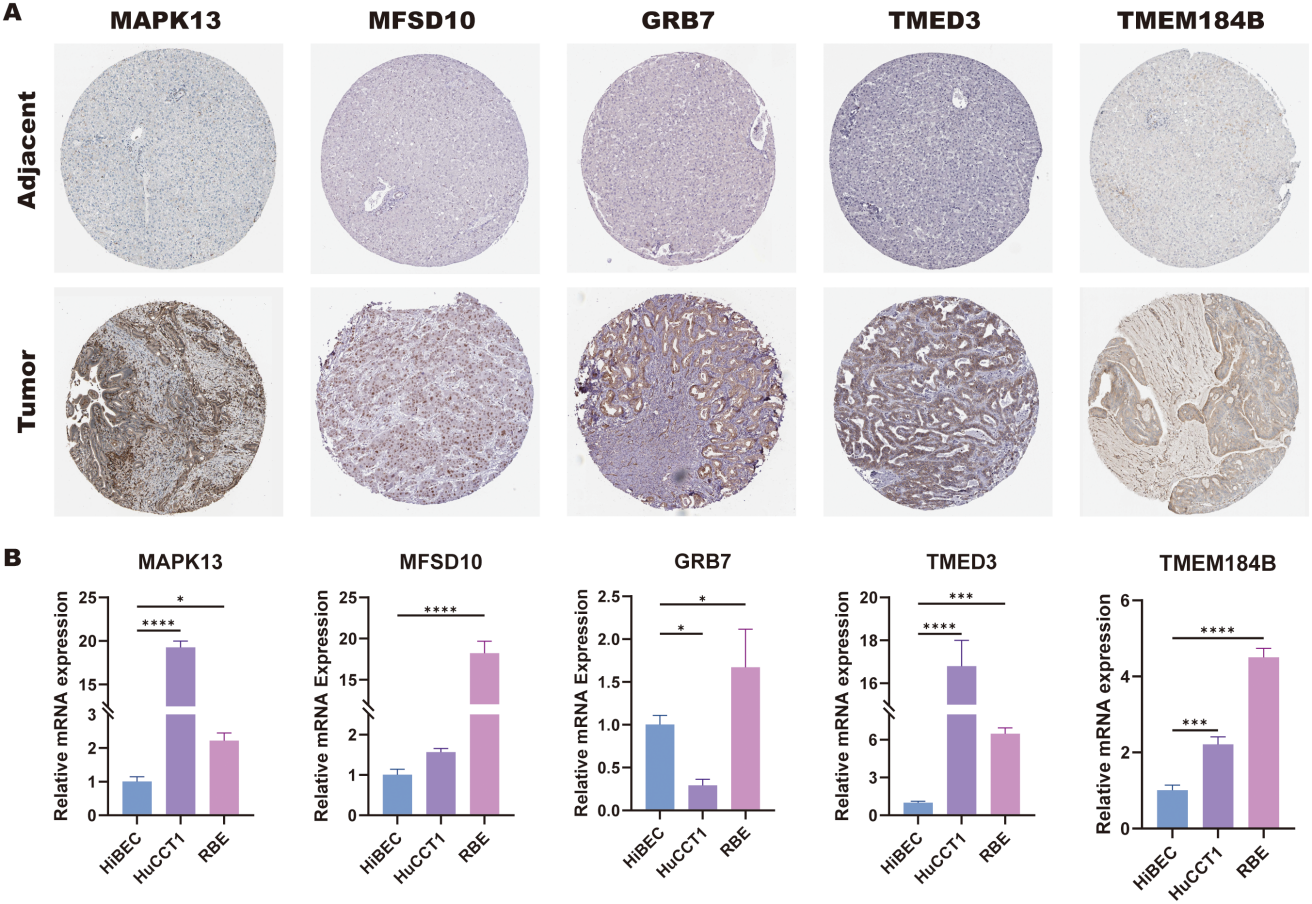
Expression validation of core genes. **(A)** Protein-level expression of core genes in cholangiocarcinoma and matched normal tissues based on data from the Human Protein Atlas **(HPA)** database. **(B)** Expression profiles of core genes in cholangiocarcinoma cell lines compared to normal cholangiocytes. Data are presented as mean ± SD. *P < 0.05, **P < 0.01, ***P < 0.001, ****P < 0.0001 vs. normal control group (one_way ANOVA followed by Dunnett's post_hoc test).

To validate the protein expression levels of the candidate genes (MAPK13, MFSD10, GRB7, TMED3, and TMEM184B), we analyzed immunohistochemistry data from the Human Protein Atlas database. The results showed that these proteins exhibited low or undetectable expression in normal liver tissues, whereas MAPK13, GRB7, and TMEM184B were markedly upregulated in intrahepatic cholangiocarcinoma tissues, with positive signals predominantly localized in the tumor cytoplasm (**Figure 9A**). MFSD10 and TMED3 exhibited moderate perinuclear staining. These protein expression patterns were consistent with the bioinformatics predictions.

To further validate the expression of the candidate genes at the transcriptional level, we performed qRT-PCR to measure the mRNA expression levels of the above genes in two cholangiocarcinoma cell lines (HuCCT1 and RBE) and normal human intrahepatic biliary epithelial cells (HiBEC). As shown in **Figure 9B**, the five candidate genes exhibited cell-line-dependent differential expression patterns in the two cholangiocarcinoma cell lines compared with HiBEC. Specifically, MAPK13, TMED3, and TMEM184B were significantly upregulated in both cancer cell lines (P < 0.01); MFSD10 showed an upward trend in HuCCT1 cells and was significantly upregulated in RBE cells; and GRB7 was upregulated in RBE cells but downregulated in HuCCT1 cells. Overall, the mRNA expression trends were largely consistent with the protein-level findings.

### 3.11 TMED3 promotes ICC cell proliferation

**Figure 10:**
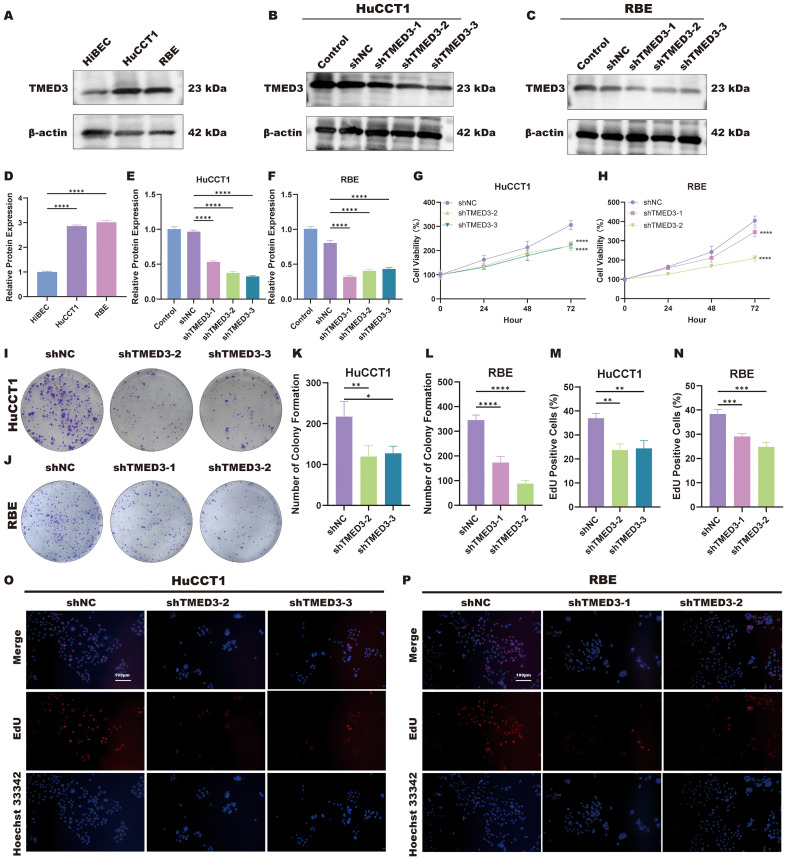
TMED3 knockdown suppresses ICC cell proliferation. **(A)** Western blot analysis of TMED3 protein expression in HiBEC, HuCCT1, and RBE cells. **(B)** Western blot validation of TMED3 knockdown efficiency in HuCCT1 cells transduced with shTMED3-carrying lentivirus. **(C)** Western blot validation of TMED3 knockdown efficiency in RBE cells transduced with shTMED3-carrying lentivirus. **(D)** Quantification of TMED3 protein expression in **(A)**. **(E)** Quantification of TMED3 knockdown efficiency in HuCCT1 cells in **(B)**. **(F)** Quantification of TMED3 knockdown efficiency in RBE cells in **(C)**. **(G)** CCK-8 assays showing the proliferation of HuCCT1 cells after TMED3 knockdown at 0, 24, 48, 72, and 96 h. **(H)** CCK-8 assays showing the proliferation of RBE cells after TMED3 knockdown at 0, 24, 48, 72, and 96 h. **(I)** Representative images of colony formation assays in HuCCT1 cells after TMED3 knockdown. **(J)** Representative images of colony formation assays in RBE cells after TMED3 knockdown. **(K)** Quantification of colony numbers in HuCCT1 cells in **(I)**. **(L)** Quantification of colony numbers in RBE cells in **(J)**. **(M)** EdU incorporation assays showing the percentage of EdU-positive cells in HuCCT1 cells after TMED3 knockdown. **(N)** EdU incorporation assays showing the percentage of EdU-positive cells in RBE cells after TMED3 knockdown. **(O)** Representative images of EdU incorporation assays in HuCCT1 cells after TMED3 knockdown. **(P)** Representative images of EdU incorporation assays in RBE cells after TMED3 knockdown. Scale bar = 100 μm. Data are presented as mean ± SD. *P < 0.05, **P < 0.01, ***P < 0.001, ****P < 0.0001 vs. NC group (two-way ANOVA for CCK-8 assays; one-way ANOVA followed by Dunnett's post-hoc test for other assays).

To examine the protein expression level of TMED3 in cholangiocarcinoma cells, we performed Western blot analysis in HiBEC, HuCCT1, and RBE cells. The results demonstrated that TMED3 was significantly upregulated in both HuCCT1 and RBE cells compared with HiBEC (**Figures 10A, D**). To investigate the functional impact of TMED3 on the malignant phenotype of ICC cells, we established stable TMED3-knockdown cell lines using lentivirus-mediated shRNA in HuCCT1 and RBE cells. Western blot analysis confirmed that all three independent shRNAs (shTMED3-1, shTMED3-2, and shTMED3-3) effectively reduced TMED3 expression in HuCCT1 cells, with shTMED3-2 and shTMED3-3 exhibiting higher knockdown efficiency (**Figures 10B, E**). Similarly, all three shRNAs effectively knocked down TMED3 in RBE cells, with shTMED3-1 and shTMED3-2 showing more pronounced effects (**Figures 10C, F**).

Based on these results, we selected the most effective shRNA constructs for subsequent functional assays: shTMED3-2 and shTMED3-3 for HuCCT1 cells, and shTMED3-1 and shTMED3-2 for RBE cells. CCK-8 proliferation assays demonstrated that TMED3 knockdown significantly suppressed the proliferative capacity of both cell lines. In HuCCT1 cells, the shTMED3-2 and shTMED3-3 groups exhibited significantly slower proliferation than the NC group from 48 h onward (**Figure 10G**). Similarly, RBE cells transfected with shTMED3-1 and shTMED3-2 showed comparable growth inhibition (**Figure 10H**).

Colony formation assays further corroborated these findings. As shown in **Figures 10I, K**, the colony numbers of HuCCT1 cells in the shTMED3-2 and shTMED3-3 groups were significantly reduced compared with the NC group (P < 0.05). Likewise, RBE cells with shTMED3-1 and shTMED3-2 knockdown formed fewer colonies than the NC group (P < 0.05, **Figures 10J, L**), indicating that TMED3 depletion impairs clonogenic growth in ICC cells.In addition, EdU incorporation assays revealed that TMED3 knockdown decreased the percentage of EdU-positive cells in both cell lines. In HuCCT1 cells, the shTMED3-2 and shTMED3-3 groups showed a significantly lower EdU-positive rate compared with the NC group (P < 0.05, **Figures 10M, O**), while in RBE cells, the shTMED3-1 and shTMED3-2 groups exhibited a similar reduction (P < 0.05, **Figures 10N, P**). Collectively, these results demonstrate that TMED3 knockdown suppresses DNA replication and proliferative capacity in ICC cells. Collectively, these findings demonstrate that TMED3 acts as a pro-proliferative driver in ICC and highlight its potential as a therapeutic target for this aggressive malignancy.

### 3.12 TMED3 promotes ICC cell migration and sensitizes cells to disulfidptosis

**Figure 11:**
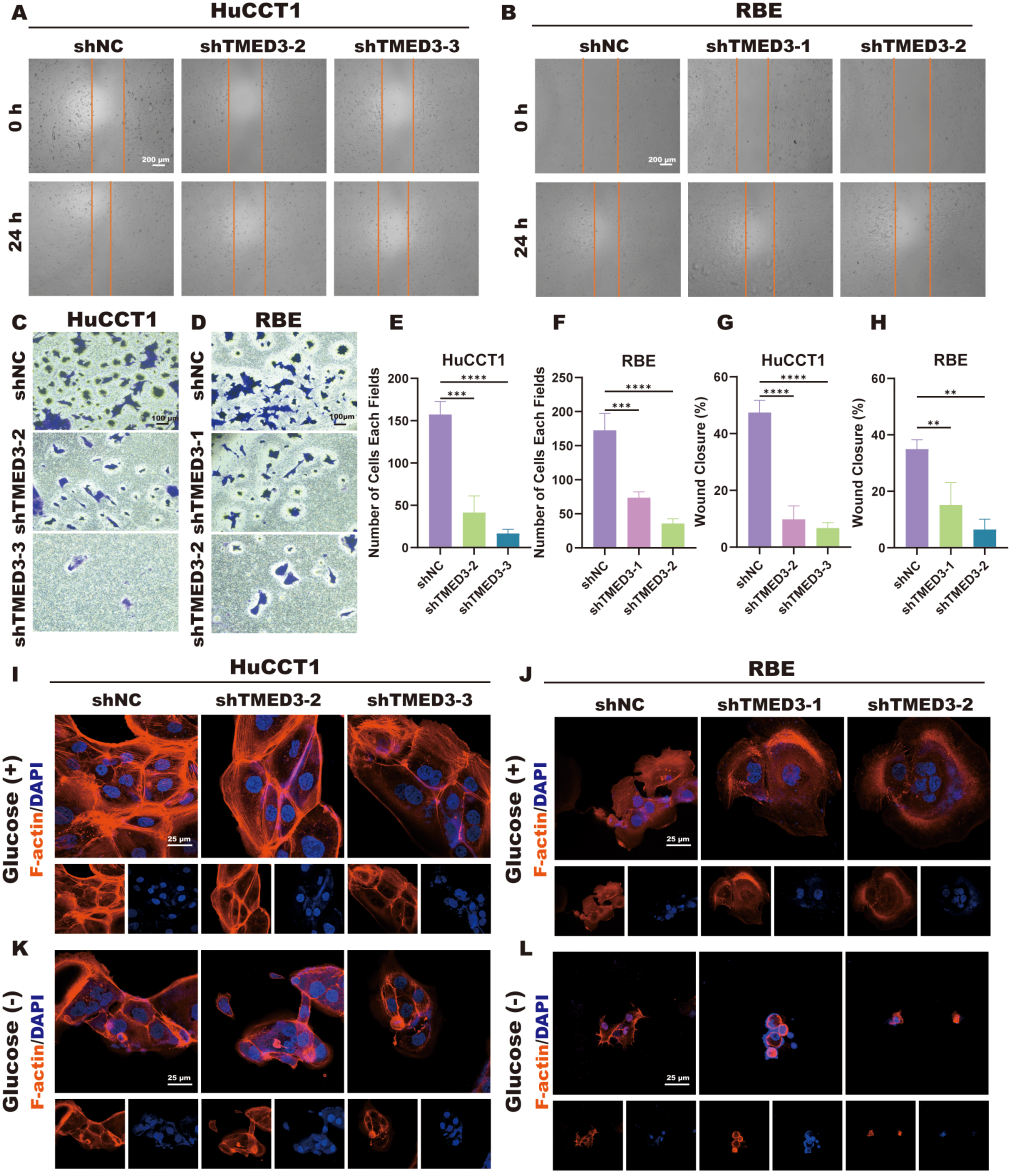
TMED3 knockdown suppresses ICC cell migration and sensitizes cells to glucose deprivation−induced disulfidptosis. **(A)** Representative images of wound healing assays showing the migratory ability of HuCCT1 cells at 0 h and 24 h after TMED3 knockdown. Scale bar = 200 μm. **(B)** Representative images of wound healing assays showing the migratory ability of RBE cells at 0 h and 24 h after TMED3 knockdown. Scale bar = 200 μm. **(C)** Representative images of Transwell migration assays showing the migratory ability of HuCCT1 cells after TMED3 knockdown. Scale bar = 100 μm. **(D)** Representative images of Transwell migration assays showing the migratory ability of RBE cells after TMED3 knockdown. Scale bar = 100 μm. **(E)** Quantification of wound closure rate in HuCCT1 cells corresponding to **(C)**. **(F)** Quantification of wound closure rate in RBE cellsÜorresponding to **(D)**. **(G)** Quantification of migrated cells per field in HuCCT1 cells corresponding to **(A)**. **(H)** Quantification of migrated cells perßield in RBE cells corresponding to **(B)**. **(I)** Representative phase_contrast images showing morphological changes of HuCCT1 cells after TMED3 knockdown cultured in normal glucose_containing medium for 24 h (control). Scale bar = 25 μm. **(J)** Representative phase_contrast images showing morphological changes of RBE cells after TMED3 knockdown cultured in normal glucose_containing medium for 24 h (control). Scale bar = 25 μm. **(K)** Representative phase_contrast images showing morphological changes of HuCCT1 cells after TMED3 knockdown cultured in glucose_free medium for 24 h to induce disulfidptosis. Scale bar = 25 μm. **(L)** Representative phase_contrast images showing morphological changes of RBE cells after TMED3 knockdown cultured in glucose_free medium for 24 h to induce disulfidptosis. Scale bar = 25 μm. Data are presented as mean ± SD. *P < 0.05, **P < 0.01, ***P < 0.001, ****P < 0.0001 vs. NC group (two_way ANOVA for wound healing assays; one_way ANOVA followed by Dunnett`s post_hoc test for Transwell migration assays).

To further evaluate the impact of TMED3 on ICC cell migration, we performed wound healing and Transwell migration assays. The wound healing assay revealed that in HuCCT1 cells, the shTMED3-2 and shTMED3-3 groups exhibited significantly slower wound closure rates compared with the NC group (**Figures 11A, G**). Similarly, in RBE cells, the shTMED3-1 and shTMED3-2 groups showed markedly reduced healing capacity (**Figures 11B, H**). The Transwell migration assay further confirmed these findings: the number of cells traversing the Transwell chambers was significantly decreased in the shTMED3-2 and shTMED3-3 groups compared with the NC group in HuCCT1 cells (**Figures 11C, E**), and a similar inhibitory trend was observed in RBE cells with shTMED3-1 and shTMED3-2 knockdown (**Figures 11D, F**). Collectively, these results demonstrate that TMED3 knockdown significantly suppresses ICC cell migration, suggesting that TMED3 plays a critical regulatory role in ICC cell migration.

Furthermore, we investigated whether TMED3 is involved in the regulation of disulfidptosis. ICC cells were cultured in glucose-free medium to induce disulfidptosis, and morphological changes were examined under a microscope. Under glucose-deprived conditions, HuCCT1 cells in the shTMED3-2 and shTMED3-3 groups exhibited more pronounced cell shrinkage, membrane blebbing, and detachment compared with the NC group (**Figures 11I, K**). Similarly, RBE cells in the shTMED3-1 and shTMED3-2 groups displayed more typical morphological features of disulfidptosis (**Figures 11J, L**). These findings suggest that TMED3 knockdown may enhance the sensitivity of ICC cells to glucose deprivation, indicating that TMED3 may participate in the regulation of disulfidptosis.

## 4 Discussion

Intrahepatic cholangiocarcinoma, the second most prevalent primary liver malignancy, is characterized by high invasiveness and marked tumor heterogeneity. Although recent advances in surgical techniques and systemic therapies have been achieved, patient outcomes remain poor, with a five-year survival rate generally below 20%, largely due to the absence of reliable early diagnostic biomarkers and effective targeted therapies (36). In this study, we systematically dissected the cellular architecture of the iCCA TME through integrated analysis of single-cell RNA sequencing and bulk transcriptomic data. We further investigated the biological properties of malignant cholangiocyte subpopulations, particularly their potential involvement in disulfidptosis—a recently identified form of regulated cell death—and developed a clinically translatable diagnostic model. Our findings provide a comprehensive cellular atlas of iCCA, identify TMED3 as a central regulatory node governing a disulfidptosis-related diagnostic signature, and offer potential biomarkers and therapeutic leads for precision oncology.

Single-cell sequencing analysis unveiled pronounced intratumoral heterogeneity in iCCA. We identified a distinct malignant cholangiocyte subpopulation exhibiting robust stemness features, which was markedly enriched in tumor tissues and displayed widespread copy number alterations. Importantly, this subpopulation demonstrated a significantly higher stemness score compared to other cellular subsets—a finding strongly aligned with the CSC paradigm, implying its potential pivotal role in tumor initiation, progression, metastasis, therapy resistance, and recurrence (37, 38). Accumulating evidence indicates that CSC-like phenotypes in iCCA are closely associated with specific surface markers, stemness-related genes, epithelial-mesenchymal transition, and altered mitochondrial activity (39, 40). Importantly, this subpopulation also exhibited significantly higher disulfidptosis activity compared with other cell types, as assessed by six independent computational algorithms. Disulfidptosis is driven by aberrant disulfide bond accumulation in actin cytoskeletal proteins, a process dependent on SLC7A11 overexpression and glucose deprivation, with NADPH depletion as the central hub (41, 42). The heightened disulfidptosis signature in malignant cholangiocytes is consistent with the well-established “Warburg effect” in iCCA, where enhanced glycolytic dependency may render these cells particularly susceptible to glucose starvation-induced disulfide stress. This metabolic vulnerability represents a promising therapeutic avenue that warrants further investigation. It should be noted, however, that these findings are primarily based on computational predictions and require experimental validation to confirm the direct involvement of disulfidptosis in iCCA pathogenesis.

To establish a mechanistic link between the five-gene diagnostic signature (TMED3, TMEM184B, MAPK13, MFSD10, and GRB7) and disulfidptosis, we performed PPI network analysis and co-expression correlation analysis. Our results demonstrated that TMED3 directly interacts with ACO2, MAPK13 interacts with ACTB and SLC2A1, and GRB7 interacts with TLN1—all of which are functionally associated with cytoskeletal organization and disulfide stress responses. Furthermore, TMED3, MAPK13, and TMEM184B showed positive correlations with core disulfidptosis regulators. These findings provide both network-level and transcriptional evidence supporting the association between the diagnostic signature and disulfidptosis-related pathways. The diagnostic and prognostic value of the signature was further validated using LASSO-logistic regression, which demonstrated robust performance in cross-validation and independent cohorts. Notably, TMED3 showed a borderline trend for PFI in multivariable analysis (HR = 2.37, P = 0.073), while TMEM184B emerged as an independent prognostic factor for DFI (HR = 4.79, P = 0.028). These results highlight the clinical potential of the signature. Nevertheless, several limitations should be considered. The small sample size of the TCGA-CHOL cohort may have limited statistical power to detect modest effect sizes, potentially underestimating the prognostic significance of some genes. Furthermore, the absence of an additional fully external cohort restricts the assessment of generalizability across diverse populations. Future studies with larger, multi-center cohorts are warranted to confirm these findings and further validate the clinical utility of the signature.

Among the five genes, TMED3 was designated as the core gene of the signature based on multiple considerations. It exhibited direct interactions with disulfidptosis-related genes in the PPI network, suggesting its potential role as a critical node in this regulatory network. Meanwhile, co-expression analysis revealed generally consistent positive correlations between TMED3 and core disulfidptosis regulators, further supporting its transcriptional coordination with this pathway. In addition, TMED3 has been documented as a pro-oncogenic factor in multiple malignancies, with well-established functions in protein trafficking and cellular stress responses that provide biological plausibility for its involvement in disulfide stress regulation (43). The signature is therefore defined as “TMED3-governed” to reflect the hierarchical role of TMED3 in bridging the diagnostic signature to disulfidptosis-related pathways, rather than implying that all five genes are directly involved in the core disulfidptosis machinery.

TMED3, a member of the p24 protein family, participates in protein trafficking between the endoplasmic reticulum and the Golgi apparatus. Accumulating evidence indicates its pro-oncogenic role in multiple solid tumors, including colorectal, hepatocellular, and esophageal carcinomas, where it is consistently associated with poor prognosis (44–47). TMED3 promotes ICC cell proliferation, migration, and disulfidptosis sensitivity. Functional experiments confirmed that TMED3 was significantly upregulated in ICC cell lines at both the protein and mRNA levels. Lentivirus-mediated knockdown of TMED3 significantly suppressed cell proliferation, colony formation, DNA synthesis, and migratory capacity in both HuCCT1 and RBE cells. Notably, under glucose-deprived conditions—a classic disulfidptosis-inducing stimulus—TMED3-knockdown cells exhibited more pronounced cell shrinkage, membrane blebbing, and F-actin disorganization, suggesting that TMED3 modulates the cellular response to disulfide stress. These findings move beyond pure bioinformatic speculation and provide direct experimental evidence supporting a functional role for TMED3 in disulfidptosis-related cellular processes. Moreover, qRT-PCR validation revealed differential expression patterns of the five candidate genes between HuCCT1 and RBE cells. The HuCCT1 cell line is derived from malignant ascites of an ICC patient and is characterized by high invasiveness and poor differentiation, whereas the RBE cell line originates from primary ICC and is relatively well-differentiated with lower invasive potential. The observed cell-line-dependent expression differences may reflect the molecular heterogeneity of ICC. These results further emphasize the necessity of validating diagnostic signatures across multiple cell lines and reflect the tumor heterogeneity of ICC.

Cell-cell communication analysis identified malignant cholangiocytes as central signaling hubs within the TME, engaging immune and stromal cells through SPP1-CD44 and IGFBP3-TMEM219 ligand-receptor pairs (48). The SPP1-CD44 axis is well-established in promoting proliferation, invasion, and immunosuppression (49), while IGFBP3/TMEM219 signaling has been implicated in hepatocyte apoptosis and fibrosis (50). Based on these known functions, our communication analysis suggests that malignant cholangiocytes may participate in TME remodeling through these pathways. Consistently, iCCA tumor tissues exhibited significantly lower stromal and ESTIMATE scores compared with adjacent normal samples. Although this observation appears counterintuitive, it is consistent with the well-documented TME heterogeneity of ICC. Large-scale TME classification studies have demonstrated that a substantial proportion of ICCs belong to stroma-poor, immune-depleted subclasses. Approximately 45% of ICCs display an immune desert phenotype characterized by downregulation of TME gene signatures (51), and the STIM classification of ~900 iCCAs revealed that non-inflamed profiles account for 65% of cases, with the desert-like class (~20%) harboring the lowest immune infiltration and the hepatic stem-like class (~35%) also showing low stromal infiltration (52). It must be emphasized that the cell-cell communication analysis presented here is entirely based on computational predictions and currently lacks experimental validation. These findings should therefore be considered exploratory and hypothesis-generating, warranting further experimental investigation to confirm their functional significance.

Based on network-based drug-target proximity analysis, nine high-priority candidate drugs were identified after excluding endogenous metabolites and restricting to FDA-approved or clinically evaluated compounds. Among these, several candidates have literature support for their potential therapeutic value in ICC or hepatobiliary malignancies. Foretinib is a multikinase inhibitor targeting MET, VEGFR2, and ROS1 (53). In a phase I/II trial as first-line therapy for advanced hepatocellular carcinoma, it demonstrated promising antitumor activity (objective response rate: 22.9%, disease control rate: 82.9%, median overall survival: 15.7 months) with a manageable safety profile, and baseline plasma IL6 or IL8 levels were suggested as potential predictors of treatment response (54). Pemetrexed is an antifolate agent that has been evaluated in biliary tract cancer clinical trials (55). A phase II study of pemetrexed plus erlotinib as salvage therapy in gemcitabine-pretreated patients with advanced biliary tract cancer showed modest clinical activity (objective response rate: 5%, disease control rate: 55%, median progression-free survival: 2.3 months, median overall survival: 5.6 months) with acceptable tolerability (56). These candidates provide preliminary directions for subsequent experimental validation. It should be emphasized that the drug prediction results presented here are exploratory in nature, and the actual efficacy of the candidate drugs requires further validation through *in vitro* and *in vivo* experiments.

This study has several limitations. First, the single−cell cohort was relatively small (8 samples, 5 tumor, 3 adjacent normal). While this sample size is comparable to several published ICC single−cell studies, the limited number of adjacent normal samples may affect the stability of the copyKAT diploid reference, and the well−recognized high inter− and intra−tumoral heterogeneity of ICC means that this cohort may not fully capture the full cellular spectrum of ICC. Therefore, our findings regarding malignant cholangiocyte aneuploidy and the cellular atlas should be interpreted as exploratory. Second, the survival analysis was constrained by the small TCGA−CHOL ICC cohort, which reduced statistical power and may have underestimated the prognostic significance of some genes, particularly TMED3 in multivariable analysis (P = 0.073). The limited sample size also warrants cautious interpretation of the high AUC values observed in our diagnostic model, and further validation in larger, independent cohorts is needed to confirm its generalizability. Third, the disulfidptosis−associated findings are based on computational prediction and preliminary morphological observations; direct molecular evidence—such as disulfide bond quantification, NADPH/NADP⁺ ratio measurements, and SLC7A11 manipulation—is needed to firmly establish the mechanistic link. Fourth, the functional experiments were limited to two ICC cell lines, and validation in additional lines and *in vivo* models is warranted. Fifth, the ESTIMATE−based TME observations may be influenced by residual bias from cross−platform batch correction, and future studies using single−platform data and orthogonal methods such as immunohistochemistry are needed to confirm the TME features observed here. Sixth, the drug prediction results are exploratory and require experimental validation. Additionally, the lack of an independent external validation cohort (e.g., from ICGC) limits the assessment of generalizability across diverse populations. Finally, the cell−cell communication analysis is entirely based on CellChat computational inference and should be considered exploratory and hypothesis−generating, with functional significance requiring further experimental validation. Despite these limitations, our study provides a comprehensive resource for understanding ICC biology and lays a foundation for future mechanistic and translational investigations.

## 5 Conclusion

In summary, by integrating multi−omics analyses, this study systematically characterized the cellular ecosystem of iCCA at single−cell resolution, identifying a malignant cholangiocyte subpopulation that co−exhibits high stemness features and susceptibility to disulfidptosis, and uncovering its distinct metabolic vulnerabilities. Among the five−gene diagnostic signature, TMED3 was identified as a key regulator linking the signature to disulfidptosis−related pathways, and its functional role in promoting ICC cell malignancy and sensitivity to disulfide stress was experimentally validated. The five−gene signature demonstrated diagnostic and prognostic promise, while network−based drug screening offered preliminary leads for therapeutic repurposing. Collectively, these findings not only advance our understanding of iCCA pathogenesis but also provide a valuable theoretical foundation and rich resource for the development of novel diagnostic tools and targeted therapeutic strategies.

## Data Availability

The datasets presented in this study can be found in online repositories. The names of the repository/repositories and accession number(s) can be found in the article/**Supplementary Material**.
