# Supplementary material for: A TMED3-governed disulfidptosis-related diagnostic signature reveals tumor microenvironment remodeling in intrahepatic cholangiocarcinoma
Source: Front Immunol. 2026 Jul 29;17:1858117. doi: 10.3389/fimmu.2026.1858117 (PMC13462420; doi:10.3389/fimmu.2026.1858117)
Supplement: Supplementary file 1 [file DataSheet1.zip › supplementary materials/supplementary materials.docx]

**Figure S1. Quality control results of doublet detection in single‑cell RNA sequencing data.** (A) UMAP visualization showing doublet predictions, with singlet cells colored in blue and predicted doublets in red; (B) density distribution of doublet scores, with the red peak representing the high‑score characteristics of the doublet population, clearly distinguishable from the singlet population (gray peak); (C) proportion of doublets in each sample, showing variability across samples (range: 0.22%–27.21%, average ~8.3%); (D) UMI count distribution of singlet and doublet cells, with doublets exhibiting significantly higher UMI counts than singlets, consistent with containing transcriptomes from two cells. Collectively, these results confirm the effectiveness of DoubletFinder‑based doublet detection, and all cells predicted as doublets were excluded from downstream analyses.

**Figure S2. Assessment of batch effect correction in bulk transcriptomic datasets.** Principal component analysis (PCA) plots showing the distribution of samples from three datasets (TCGA, GSE107943, and GSE32225) before (A) and after (B) ComBat batch correction. Each dot represents an individual sample, and colors indicate the dataset origin (TCGA: orange; GSE107943: red; GSE32225: blue). Prior to correction (A), samples from the three datasets exhibited clear separation along the PC1 and PC2 axes, indicating substantial batch effects. After ComBat correction (B), samples from different datasets became well‑intermixed, demonstrating effective removal of batch effects while preserving biological variation between tumor and adjacent normal samples.

**Figure S3. Bubble plot of disulfidptosis‑related pathway activity scores calculated by the singscore algorithm.** Rows represent seven cell subpopulations of the liver microenvironment, and columns represent various classical functional and signaling pathways. Bubble color indicates the direction of pathway activity changes (blue: log₂FC < 0, downregulated; red: log₂FC > 0, upregulated), and bubble size corresponds to −log₁₀(adjusted P‑value), with larger bubbles reflecting higher statistical significance of pathway activity differences between tumor and adjacent normal cells. The plot illustrates the differential activity patterns of each pathway across distinct cell subpopulations between tumor and paired adjacent normal tissues.

**Figure S4. Bubble plot of disulfidptosis‑related pathway activity scores calculated by the ssGSEA algorithm.** Rows represent seven cell subpopulations of the liver microenvironment, and columns represent various classical functional and signaling pathways.

**Figure S5. Bubble plot of disulfidptosis‑related pathway activity scores calculated by the viper algorithm.** Rows represent seven cell subpopulations of the liver microenvironment, and columns represent various classical functional and signaling pathways.

**Figure S6. Decision curve analysis and calibration curve for the five-gene diagnostic signature in the TCGA-CHOL ICC cohort.** **(A)** Decision curve analysis (DCA) evaluating the clinical net benefit of the five‑gene diagnostic signature across a range of threshold probabilities in the TCGA‑CHOL intrahepatic cholangiocarcinoma (ICC) cohort. **(B)** Calibration curve assessing the agreement between predicted probabilities and observed outcomes for the five‑gene signature in the TCGA‑CHOL ICC cohort. The x‑axis represents the predicted probability of tumor, and the y‑axis represents the observed proportion.
